# DNA Replication Control Is Linked to Genomic Positioning of Control Regions in *Escherichia coli*

**DOI:** 10.1371/journal.pgen.1006286

**Published:** 2016-09-02

**Authors:** Jakob Frimodt-Møller, Godefroid Charbon, Karen A. Krogfelt, Anders Løbner-Olesen

**Affiliations:** 1 Department of Biology, Section for Functional Genomics and Center for Bacterial Stress Response and Persistence (BASP), University of Copenhagen, Copenhagen, Denmark; 2 Department of Microbiology and Infection Control, Statens Serum Institut, Copenhagen, Denmark; University of Geneva Medical School, SWITZERLAND

## Abstract

Chromosome replication in *Escherichia coli* is in part controlled by three non-coding genomic sequences, *DARS1*, *DARS2*, and *datA* that modulate the activity of the initiator protein DnaA. The relative distance from *oriC* to the non-coding regions are conserved among *E*. *coli* species, despite large variations in genome size. Here we use a combination of i) site directed translocation of each region to new positions on the bacterial chromosome and ii) random transposon mediated translocation followed by culture evolution, to show genetic evidence for the importance of position. Here we provide evidence that the genomic locations of these regulatory sequences are important for cell cycle control and bacterial fitness. In addition, our work shows that the functionally redundant *DARS1* and *DARS2* regions play different roles in replication control. *DARS1* is mainly involved in maintaining the origin concentration, whether *DARS2* is also involved in maintaining single cell synchrony.

## Author Summary

Replication of the *E*. *coli* chromosome is the central event in the cell cycle, with the control of replication enforced at the level of initiation. DnaA is the key protein responsible for initiation at the origin of replication (*oriC*), and is active in this when bound to ATP and inactive when bound to ADP. The activity of DnaA is in part controlled by three non-coding DNA regions; *datA*, *DARS1*, and *DARS2*. Here we show that the chromosomal position of *datA*, *DARS1*, and especially *DARS2* relative to *oriC*, is important for cell cycle control and bacterial fitness. Based on this and previous work, we propose that the functionally redundant *DARS1* and *DARS2* regions play different roles in replication control. *DARS1* is mainly involved in maintaining the origin concentration, while *DARS2* is also involved in maintaining single cell synchrony. Both regions are needed for proper replication control, and this also provides an explanation for the conservation observed in all sequenced *E*. *coli* strains. Further, the present literature indicates that these observations can be applied to other related bacteria.

## Introduction

The circular chromosome of *Escherichia coli* is replicated bidirectionally from a single origin, *oriC*. The DnaA protein is responsible for replication initiation [1]. DnaA belongs to the AAA^+^ (ATPases Associated with diverse Activities) proteins and can bind both ATP and ADP with similar high affinities[1]. DnaA is active in replication when bound to ATP (DnaA^ATP^)[2] and facilitates the unwinding of *oriC* [3–5]. Integration Host Factor (IHF) [6] and DiaA [7, 8] stimulates initiation from *oriC*, while initiation is opposed by the binding of Fis to *oriC* [9–11]. After open complex formation, DnaA loads the DnaB helicase onto the single-stranded DNA, which promotes further duplex opening and assembly of the replisome [5]. After initiation DnaA^ATP^ is inactivated, i.e. converted to DnaA^ADP^, by RIDA (Regulatory Inactivation of DnaA) [12–14] and DDAH (*datA*-dependent DnaA^ATP^ hydrolysis) [15] to prevent re-initiation. RIDA is more efficient in lowering the DnaA^ATP^/DnaA^ADP^ ratio than DDAH [15]. At later cell cycle stages DnaA^ADP^ is reactivated at the two DnaA-Reactivating Sequences (*DARS1* and *DARS2*) to allow for the next round of initiation [16, 17]. *DARS1* is not regulated by any known pathway [16], while *DARS2* activity is modulated by both an IHF- and Fis-dependent pathway [17].

In *E*. *coli*, there is a selective pressure to maintain chromosome symmetry; i.e. two nearly equal length replication arms [18]. The *datA*, *DARS1*, and *DARS2* regions have the same relative distance to *oriC* in all *E*. *coli* strains sequenced [19], but none of the loci alone or in combination are essential for cell viability. Loss of either region is however associated with a fitness cost [19].

The *E*. *coli* chromosome consist of four insulated macrodomains (MD) and two less constrained regions called nonstructured (NS) regions [20, 21]. DNA recombination occurs preferentially within MDs, while DNA interactions between the different MDs are highly restricted. The two NS regions can however interact with both its flaking MDs [21]. The Ori MD is flanked by the two NS (NS^Right^ and NS^Left^) whereas the Ter MD is flanked by the Left and Right MDs [21]. Both *oriC* and *datA* are located within the Ori MD, *DARS2* within the NS^Left^, *DARS1* within the Right MD, and *terC* in the Ter MD [19]. Chromosomal rearrangements resulting in a mixture of different macrodomains have deleterious effects of cell growth whereas rearrangements within them are better tolerated [22].

Chromosome organization is reported to affect gene expression, mainly at the transcriptional level. A recent study found that a reporter gene cassette, comprised of the *lac* promoter driving expression of *gfp*, varied ∼300-fold depending on its position on the chromosome, in a manner fairly unrelated to the replication-associated gene dosage [23]. Gene expression even varies between insertion sites within the same MD, and both MD- and NS-regions contain high and low activity regions [23], due to intrinsic properties of the region. However, for fast growing bacteria the replication-associated gene dosage determines the organization of the chromosome; i.e. genes involved in translation and transcription (but not other highly expressed genes) are located close to *oriC* [24]. The selective pressure that keeps these genes in the relative proximity to *oriC* could be due to the challenges that *E*. *coli* faces a very high growth rates, which are hardly observable at lower growth rates [24]. The replication-associated gene dosage could also be important for the activity of non-coding regions such as *datA*, *DARS1*, and *DARS2*.

Here we show that the genomic location of *datA*, *DARS1*, and *DARS2* are important for cell cycle control and for bacterial fitness. This provides a direct link between DNA replication control and genomic positioning.

## Results

### Chromosomal position of *datA*, *DARS1*, and *DARS2*

**Fig 1:**
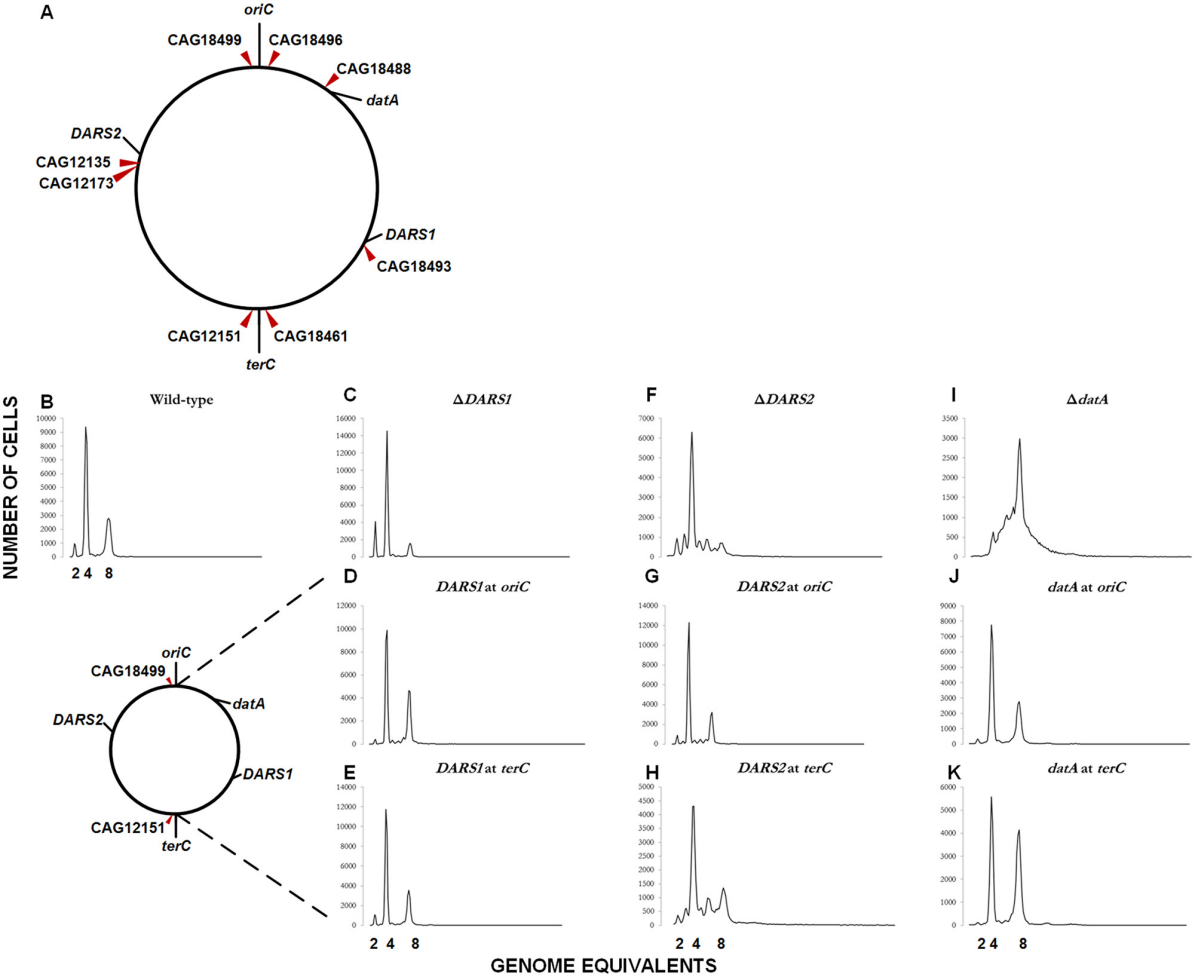
Site specific relocation of *datA*, *DARS1* and *DARS2*. (A) A schematic presentation of *oriC*, *datA*, *DARS1*, *DARS2*, *terC*, and the chosen Tn*10* positions on the MG1655 chromosome. The MG1655 chromosome is shown as a circle and the locations of *datA* (at the genome map position of 94.6 min), CAG18488 (at 93.9 min), CAG18496 (at 86.8 min), *oriC* (at 84.6 min), CAG18499 (at 83.5 min), *DARS2* (at 64.0 min), CAG12135 (at 63.6 min), CAG12173 (at 62.2 min), CAG12151 (at 38.3 min), *terC* (around 36 min), CAG18461 (at 33.3 min), CAG18493 (at 17.7 min), and *DARS1* (at 17.5 min) are indicated. CAG18496 and CAG18499 are chosen to represent *oriC*, CAG18488 to represent *datA*, CAG12135 and CAG12173 to represent *DARS2*, CAG18493 to represent *DARS1*, and CAG18461 and CAG12151 to represent *terC*. Figure is not to scale. (B-K) Representative flow cytometry histograms of a Tn*10* insertion close to *oriC* (CAG18499) and one close to *terC* (CAG12151). Prior to flow cytometric analysis exponentially growing wild-type and mutant cells was treated with rifampicin and cephalexin. Cells were grown in AB minimal medium supplemented with 0.2% glucose, 10 μg/ml thiamine, and 0.5% casamino acids at 37°C. Wild-type, Δ*DARS1*, Δ*DARS2*, and Δ*datA* are shown in B, C, F, and I, respectively. Derivatives of the wild-type strain MG1655 devoid of *DARS1*, *DARS2*, or *datA* at the original locus and instead carrying Tn*10*::*DARS1* (D and E), Tn10::*DARS2* (G and H), or Tn*10*::*datA* (J and K), respectively, at indicated chromosomal loci are shown in individual panels. See Table 1 for details.

The conserved relative distances from *oriC* to the *DARS1*, *DARS2* and *datA* regions in *E*. *coli* [25], suggest that their chromosomal positions are important for correctly controlled replication initiation. In order to construct strains carrying *datA*, *DARS1*, and *DARS2* at different loci, eight Tn*10* insertions from the Singer library were chosen [26, 27] (Fig 1A). Strains had their respective chromosomal *datA*, *DARS1*, or *DARS2* loci deleted, and the *tetC* gene of Tn*10* replaced with the *datA*, *DARS1*, or *DARS2* loci respectively, resulting in strains carrying a single copy of the respective region at a new chromosomal location. Mutant strains were evaluated by growth- and flow cytometry studies (Materials and Methods).

Wild-type cells exhibited the expected synchronous initiation pattern with the majority of cells containing 2, 4 or 8 replication origins (Fig 1B). Cells deficient in *datA* have an increase in the DnaA^ATP^/DnaA^ADP^ ratio [15, 28], resulting in an increased origins/mass, and a high degree of initiation asynchrony (Fig 1I) [25]. Rifampicin-resistant initiations, that could be suppressed by increasing the drug concentration, have previously been reported for cells deficient in *datA* [29]. However, a fourfold increased rifampicin concentration did not affect any of the parameters measured here. Relocating *datA* to any of the positions close to *oriC* (CAG18499 or CAG18496), *datA* (CAG18488), or *DARS2* (CAG12173) resulted in cell cycle parameters similar to wild-type (Table 1). Relocation to the positions near *DARS1* (CAG18493) or especially *terC* (CAG12151 and CAG18461) resulted in an increase in origin concentration but no asynchrony (Table 1; Fig 1K). This suggests that although *datA* is functional when located close to the termini the cells initiate replication at a decreased initiation mass relative to wild-type. This correlates with previous observations [28]. To test if the reduced *datA* function in the terminus region was related to gene dosage we integrated a 2^nd^ *datA* region close to *terC* (Δ*datA* CAG12151::*datA* CAG18461::*datA*; Table 1). A cell with two *datA* regions close to *terC* has an origin concentration slightly below wild-type, but initiates initiation in synchrony. The chromosomal wild-type position of *datA* has a gene dosage of 2, while the Tn*10* positions CAG12151 and CAG18461 have gene dosages of 1.1 and 1.0, respectively (Materials and Methods). This suggests that the copy number is important for correct *datA* function; a single *datA* relocated to *terC* has a reduced function, which can be complemented by a second *datA* in *terC* that brings the overall *datA* copy number up to wild-type level.

**Table 1:** Effect of different chromosomal positions for *datA*, *DARS1*, *DARS2* in MG1655.

Strains	RD to *oriC*^a^	DT^b^	Origin/mass^c^	Asynchrony index^d^
Wild-type		40 min	1	0.05
Δ*datA*	0.10	41 min	1.25 (0.05)	0.40
Δ*DARS1*	0.33	40 min	0.82 (0.01)	0.06
Δ*DARS2*	0.21	41 min	0.82 (0.01)	0.30
Δ*datA* CAG18499::*datA*::*cat*	0.01	36 min	0.98 (0.01)	0.06
Δ*datA* CAG18496::*datA*::*cat*	0.02	37 min	0.97 (0.02)	0.06
Δ*datA* CAG18488::*datA*::*cat*	0.09	40 min	0.97 (0.04)	0.06
Δ*datA* CAG12135::*datA*::*cat*	0.21	ND	ND	ND
Δ*datA* CAG12173::*datA*::*cat*	0.22	39 min	1.01	0.06
Δ*datA* CAG18493::*datA*::*cat*	0.33	38 min	1.06 (0.01)	0.05
Δ*datA* CAG18461::*datA*::*cat*	0.48	40 min	1.12 (0.02)	0.06
Δ*datA* CAG12151::*datA*::*cat*	0.46	38 min	1.09 (0.01)	0.06
Δ*datA* CAG12151::*datA*::*cat* CAG18461::*datA*		43 min	0.95 (0.04)	0.08
Δ*DARS1* CAG18499::*DARS1*::*cat*	0.01	41 min	1.10 (0.04)	0.09
Δ*DARS1* CAG18496::*DARS1*::*cat*	0.02	38 min	1.09 (0.03)	0.08
Δ*DARS1* CAG18488::*DARS1*::*cat*	0.09	40 min	1.03 (0.01)	0.06
Δ*DARS1* CAG12135::*DARS1*::*cat*	0.21	40 min	1.01 (0.04)	0.09
Δ*DARS1* CAG12173::*DARS1*::*cat*	0.22	40 min	0.99	0.09
Δ*DARS1* CAG18493::*DARS1*::*cat*	0.33	43 min	0.96 (0.02)	0.08
Δ*DARS1* CAG18461::*DARS1*::*cat*	0.48	40 min	0.97 (0.02)	0.06
Δ*DARS1* CAG12151::*DARS1*::*cat*	0.46	42 min	0.97 (0.00)	0.08
Δ*DARS2* CAG18499::*DARS2*::*cat*	0.01	41 min	0.99 (0.04)	0.10
Δ*DARS2* CAG18496::*DARS2*::*cat*	0.02	40 min	1.02 (0.04)	0.10
Δ*DARS2* CAG18488::*DARS2*::*cat*	0.09	40 min	0.99 (0.01)	0.10
Δ*DARS2* CAG12135::*DARS2*::*cat*	0.21	42 min	1.01 (0.02)	0.08
Δ*DARS2 yafJ*::*DARS2*::*cat*	0.21	40 min	1.00	0.11
Δ*DARS2* CAG12173::*DARS2*::*cat*	0.22	ND	ND	ND
Δ*DARS2* CAG18493::*DARS2*::*cat*	0.33	42 min	0.98 (0.01)	0.10
Δ*DARS2* CAG18461::*DARS2*::*cat*	0.48	42 min	0.95 (0.05)	0.27
Δ*DARS2* CAG12151::*DARS2*::*cat*	0.46	42 min	0.96 (0.04)	0.41

^a^ Relative distance from *oriC* to insertion (see Materials and Methods).

^b^ Doubling time in minimal medium supplemented with glucose and casamino acids grown at 37°C

^**c**^ Determined as average light scatter from flow cytometric analysis. Numbers are normalized to 1 for wild-type. Standard error is indicated in the brackets.

^d^ Asynchrony index; calculated as described in Materials and Methods.

Cells deficient in *DARS1* (Fig 1C) or *DARS2* (Fig 1F) have a reduced DnaA^ATP^/DnaA^ADP^ ratio compared to wild-type [16, 17]; resulting in a reduced origin concentration compared to wild-type and our data are in agreement with this. Asynchrony of initiation was only observed in Δ*DARS2*, but not in Δ*DARS1* [16].

The chromosomal position of *DARS1* did not affect cellular doubling time or single cell initiation synchrony (Table 1). When *DARS1* was relocated from its normal position to one of the two positions bordering *oriC*, the cells contained an elevated number of origins and an increased origin concentration (Fig 1D). We propose that *DARS1* located close to *oriC* results in a phenotype similar to *datA* relocated to the termini (see above), where cells initiate replication at a decreased initiation mass relative to wild-type.

Relocation of *DARS2* influenced cell cycle parameters to a larger extent than *DARS1*. A wild-type phenotype was obtained for the five locations, CAG18499, CAG18496, CAG18488, CAG12135, and CAG18493 (Table 1). However when *DARS2* were moved to positions close to *terC*, cells initiated asynchronously and had a decreased origin concentration relative to wild-type, although less than that of a *DARS2* deletion (Fig 1H); indicating a still somewhat functional *DARS2* in the terminus. In addition we tested the relocation of *DARS2* to *yafJ*, a putative glutamine amidotransferase, located with the same relative distance to *oriC* as the wild-type *DARS2* position, but on the other replication arm. Here *DARS2* had a similar doubling time, origin concentration, and synchrony as the wildtype (Table 1).

In order to assess whether the replication defect of cells carrying *DARS2* near *terC* resulted solely from a gene dosage effect we cloned *DARS2* into the F-based plasmid pALO277 [30]. The F plasmid has a copy number of 1–2 plasmids per genome equivalent [31], which is equal to or higher than the copy number of *DARS2* at its chromosomal location (Materials and Methods). Replication of the F plasmid is limited by the availability of RepE [32], but DnaA is also required [33]. To ensure that the presence of *DARS2* on pALO277 did not alter the copy number we determined the number of the F plasmid relative to the termini by qPCR analysis for the wild-type cell transformed with either pALO277 or pALO277::*DARS2 (*S1 Fig). The data shows that the presence of *DARS2* on the F plasmid does not alter the copy number. Wild-type cells with pALO277::*DARS2* showed an increased origin/mass, indicating a functional *DARS2* locus on pALO277::*DARS2* (S1 Fig). Δ*DARS2* cells were only partly complemented by pALO277::*DARS2* as their origin concentration remained below wild-type level and initiation synchrony was never restored. These observations suggest that the contribution of *DARS2* to cell cycle control is not solely through copy number. As plasmid F replicates randomly in the cell cycle [34] the coordination of *DARS2* replication relative to *oriC* and other *cis*-acting control regions is no longer present and this may explain why *DARS2* deficiency cannot be complemented by a plasmid borne *DARS2* copy.

### Importance of having both *DARS1* and *DARS2*

*DARS1* and *DARS2* both rejuvenate inactive DnaA^ADP^ to active DnaA^ATP^ [16, 17]. We proceeded to investigate if loss of either locus could be complemented by an extra copy of the other. The effect of having two functional *DARS2* loci were investigated in a Δ*DARS1* cell, i.e., retained the original wild-type *DARS2* locus, with the addition of a *DARS2* inserted into one of seven Tn*10* positions.

Loss of *DARS1* did not result in initiation asynchrony, and an extra copy of *DARS2* only changed this when inserted close to *oriC* in CAG18499 (although only marginally with and asynchrony index of 0.17) but not in any of the remaining tested positions (Table 2). Why a 2^nd^ copy of *DARS2* in CAG18499 but not CAG18496 gives asynchrony is unknown. *DARS2* reintroduced close to the termini, close to the wildtype *DARS2* position and even close to *datA*, which is located near *oriC*, failed to fully complement loss of *DARS1* with respect to origin concentration (Table 2; S2 Fig). An additional copy of *DARS2* only fully complemented *DARS1* deficiency, i.e. with respect to origin concentration and synchrony, when introduced at a position close to *oriC* (CAG18496) or precisely at the wild-type *DARS1* position (replacing the chromosomal *DARS1* copy with a 2^nd^ chromosomal *DARS2* copy). This suggests that a relatively high gene dosage of *DARS2* is required to complement *DARS1* deficiency and that the context of the *DARS1* region might be favorable for rejuvenation of DnaA^ADP^ to DnaA^ATP^, hence the 2^nd^ *DARS2* copy here can fully complement to wild-type even though the chromosomal position does not provide a high copy number.

**Table 2:** Effect of having only two functional *DARS1* or *DARS2* loci.

Strains	RD to *oriC*^a^	DT^a^	Origin/mass^b^	Asynchrony index^c^
Wild-type		40 min	1	0.05
Δ*DARS1*	0.33	40 min	0.82 (0.01)	0.06
Δ*DARS2*	0.21	41 min	0.82 (0.01)	0.30
Δ*DARS1* CAG18499::*DARS2*::*cat*	0.01	42 min	1.02 (0.03)	0.17
Δ*DARS1* CAG18496::*DARS2*::*cat*	0.02	41 min	1.02 (0.00)	0.08
Δ*DARS1* CAG18488::*DARS2*::*cat*	0.09	39 min	0.92 (0.04)	0.08
Δ*DARS1* CAG12135::*DARS2*::*cat*	0.21	40 min	0.80	0.08
Δ*DARS1* CAG12173::*DARS2*::*cat*	0.22	38 min	0.86	0.08
Δ*DARS1* CAG18493::*DARS2*::*cat*	0.33	41 min	ND	ND
Wild-type *DARS1*::*DARS2*::*cat*	0.33	36 min	1.00 (0.00)	0.10
Δ*DARS1* CAG18461::*DARS2*::*cat*	0.48	40 min	0.93 (0.03)	0.07
Δ*DARS1* CAG12151::*DARS2*::cat	0.46	39 min	0.93	0.06
Δ*DARS2* CAG18499::*DARS1*::*cat*	0.01	38 min	1.05 (0.01)	0.52
Δ*DARS2* CAG18496::*DARS1*::*cat*	0.02	42 min	1.06	0.27
Δ*DARS2* CAG18488::*DARS1*::*cat*	0.09	39 min	0.99 (0.00)	0.44
Δ*DARS2* CAG12135::*DARS1*::*cat*	0.21	38 min	1.00 (0.00)	0.28
Δ*DARS2* CAG12173::*DARS1*::*cat*	0.22	37 min	0.99	0.28
Δ*DARS2* CAG18493::*DARS1*::*cat*	0.33	42 min	1.09 (0.07)	0.65
Δ*DARS2* CAG18461::*DARS1*::*cat*	0.48	41 min	0.97	0.50
Δ*DARS2* CAG12151::*DARS1*::*cat*	0.46	39 min	0.97 (0.00)	0.49

^a^ Relative distance from *oriC* to transposon insertion (see Materials and Methods).

^b^ Doubling time in minimal medium supplemented with glucose and casamino acids grown at 37°C

^**c**^ Determined as average light scatter from flow cytometric analysis. Numbers are normalized to 1 for wild-type. Standard error is indicated in the brackets.

^d^ Asynchrony index; calculated as described in Materials and Methods.

The ability of *DARS1* to complement *DARS2* deficiency was addressed by the same approach. The decrease in origin concentration observed for *DARS2* deficient cells could be fully complemented by an additional *DARS1* copy, irrespective of chromosomal position (Table 2; S2 Fig), although slightly elevated when *DARS1* were reintroduced close to *oriC*. However, the additional *DARS1* locus failed in all cases to complement the asynchrony phenotype of *DARS2* deficient cells. These data suggests that *DARS2* is a poor replacement for *DARS1* and vice visa. They also indicate that *DARS1* and *DARS2* serves different functions that are both required for an efficient control of the cell cycle.

### Optimal chromosomal position of *DARS1* and *DARS2* during fast growth

The optimal chromosomal positions of *DARS1* and *DARS2* were determined by in-culture evolution using a novel transposon mediated approach. Here, the chromosomal *DARS1* and *DARS2* loci were cloned into the mini Tn*10* based transposon NKBOR (on pNKBOR) [35], resulting in NKBOR::*DARS1* (pJFM3) and NKBOR::*DARS2* (pJFM1), respectively (Materials and Methods). pNKBOR is a R6K-based suicide vector [35]. Hence when pJFM1 or pJFM3 were transformed into the Pir deficient strain MG1655, random insertions of either NKBOR::*DARS1* or NKBOR::*DARS2* were obtained; these are for simplicity referred to as *DARS1* or *DARS2* insertions, respectively. Five different experiential set-ups were performed; NKBOR into wild-type, *DARS1* into Δ*DARS1* and Δ*DARS1* Δ*DARS2* cells, and *DARS2* into Δ*DARS2* and Δ*DARS1* Δ*DARS2* cells, where an estimated 70,000 different transposon insertions were obtained for each, corresponding to an insert pr. 65 base pairs. We feel this is more than adequate to evaluate the importance of the genomic position for the non-coding regions.

**Fig 2:**
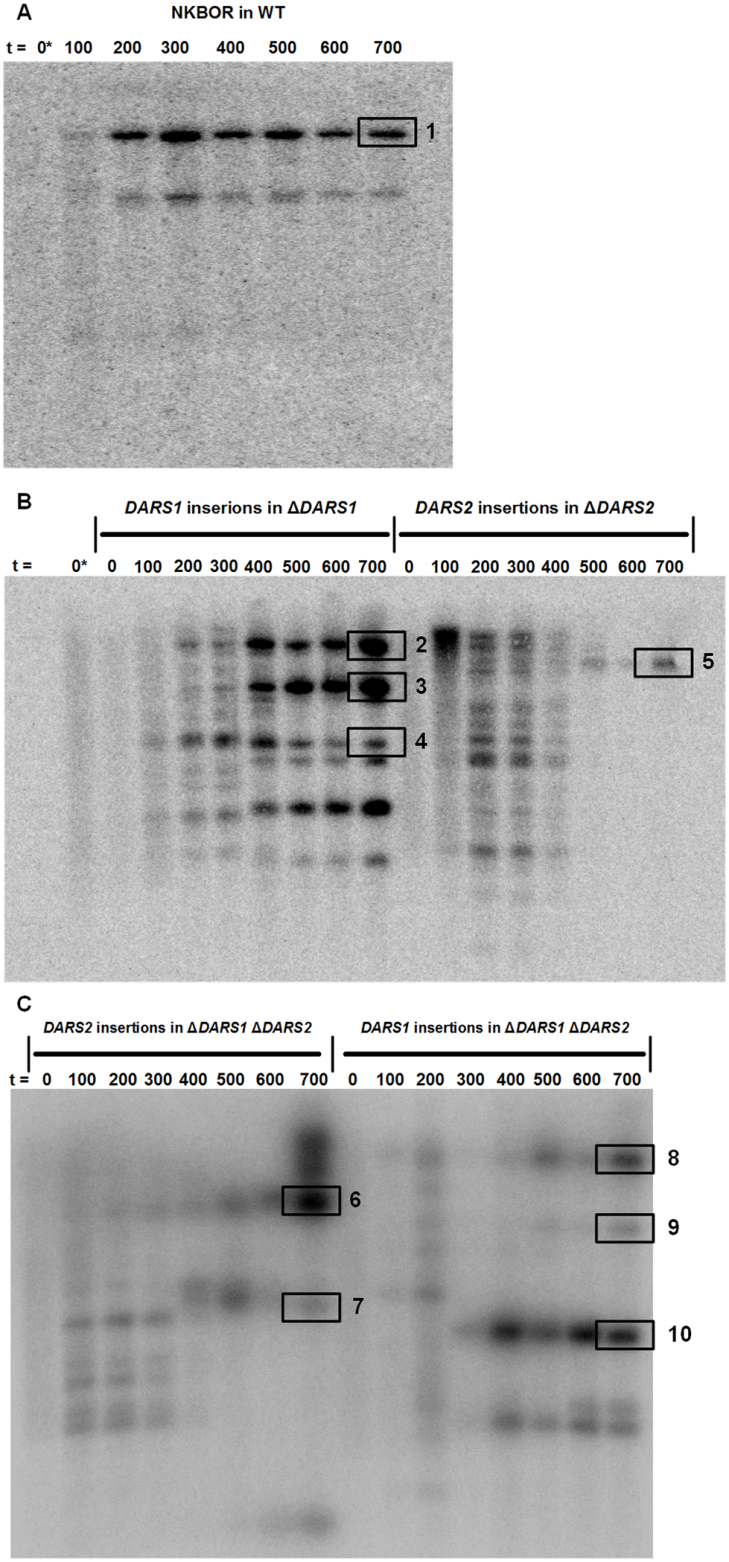
Southern blot probed for NKBOR. t shows number of estimated generations of direct competition. 0* is NKBOR into wild-type at t = 0 in both 3A and 3B. 3A shows NKBOR into wild-type, 3B shows *DARS1* insertion into *DARS1* deficient cells and *DARS2* into *DARS2* deficient cells, while 3C shows *DARS1* into *DARS1 DARS2* deficient cells and *DARS2* into *DARS1 DARS2* deficient cells. Selected representative bands are highlighted: 1. NKBOR Clone *fimE* (#1–#4); 2. *DARS1* Clone *fimE*; 3. *DARS1* Clone *yfb*; 4. *DARS1* Clone *ydeS*; 5. *DARS2* Clone *rrpH/IR*; 6. *DARS2* Clone *lgo*/*lgoR*; 7. *DARS2* Clone *ttdR*; 8. *DARS1* Clone *fimE* (#5-#6); 9. *DARS1* Clone *tomB*; 10. *DARS1* Clone *gsp*. See Materials and Methods for details.

The 70,000 different insertions were pooled for each set-up (t = 0) and continuously propagated in LB for a total of 700 generations (t = 700). The hypothesis is that the optimal position of *DARS1*/*DARS2* would result in the fittest clone, which over time would out-compete the rest. A Southern Blot, probed for NKBOR, was performed to confirm that the output of the direct competition experiment would become more clonal over time (Fig 2). Representative insertion sites from the Input, selected time points and end (t = 700) transposon pools were mapped by full genome sequencing (Materials and Methods). In addition, single clones were isolated after 700 generations from each set-up, for further investigation, and their precise transposon locations were determined by easy gene walking ([36]; Materials and Methods).

**Fig 3:**
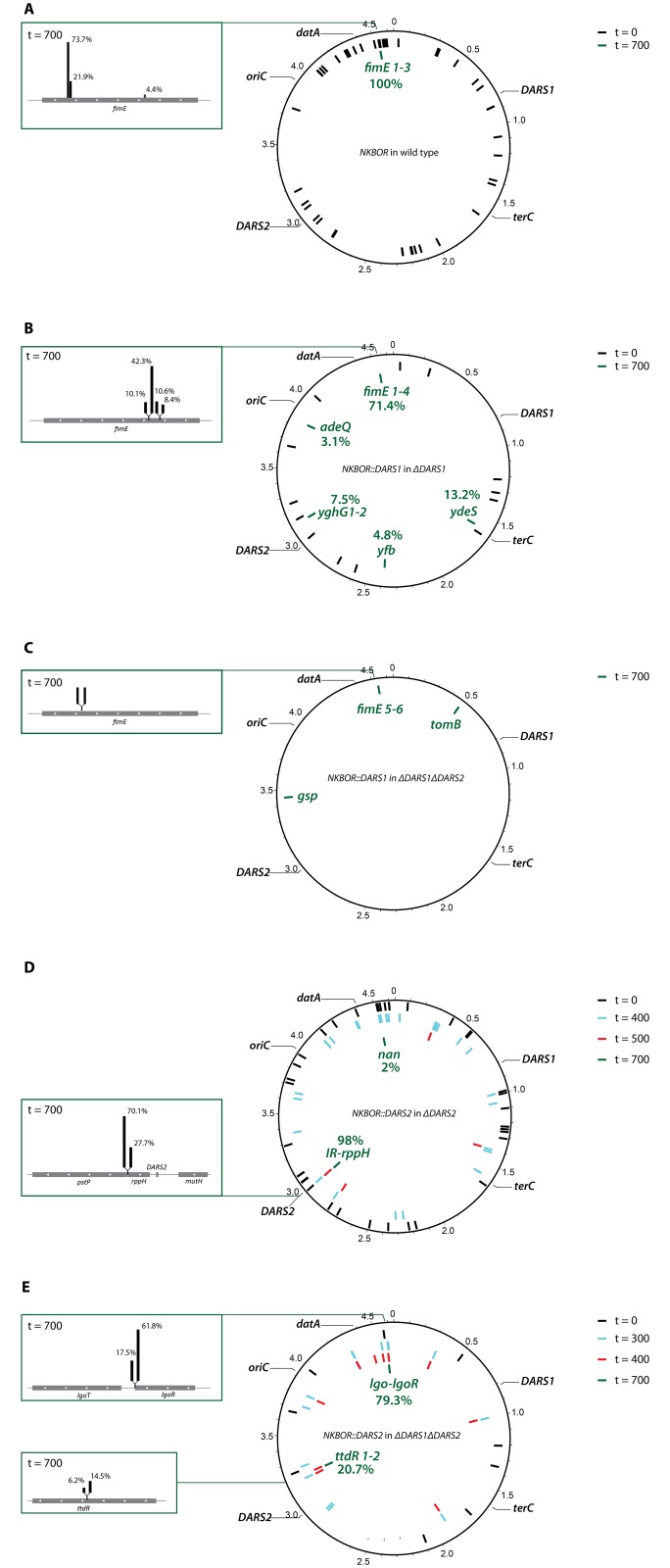
Graphic representation of resolved transposon insertions sites in wild-type, Δ*DARS1*, Δ*DARS2*, and Δ*DARS1* Δ*DARS2*. The position of *oriC*, *datA*, *DARS1*, *DARS2*, and *terC* is indicated in the individually figures. The number of insertion sites is displayed in percent for insertions resolved at t = 700. (A) NKBOR insertion sites in the wild-type at t = 0 (black bars) and t = 700 (green bars) resolved by full genome sequencing. Exact insertions sites are listed in S1 Table. (B) *DARS1* insertion sites in Δ*DARS1* at t = 0 (black bars) and t = 700 (green bars) resolved by full genome sequencing. Exact insertions sites are listed in S2 Table. (C) *DARS1* insertion sites in Δ*DARS1* Δ*DARS2* at t = 700 (green bars) resolved by easy gene walking. Exact insertions sites are listed in S3 Table. (D) *DARS2* insertion sites in Δ*DARS2* at t = 0 (black bars), t = 400 (light blue bars), t = 500 (red bars) and t = 700 (green bars) resolved by full genome sequencing. Exact insertions sites are listed in S4 Table. (E) *DARS2* insertion sites in Δ*DARS1* Δ*DARS2* at t = 0 (black bars), t = 300 (light blue bars), t = 400 (red bars) and t = 700 (green bars) resolved by full genome sequencing. Exact insertions sites are listed in S5 Table. Figure was made using DNAPlotter [67].

The mini-transposon NKBOR (Fig 2A), *DARS1 (*Fig 2B and 2C), and *DARS2 (*Fig 2B and 2C) were as expected inserted randomly throughout genome at t = 0. After 700 generations of growth, all NKBOR insertions into wild-type cells were mapped to three locations in the *fimE* gene (termed NKBOR Clone *fimE* #1, #2, and #3, Fig 3A, S1 Table). This indicates that under the used experimental settings the disruption of *fimE* resulted in a fitness advantage. Our Southern Blot showed two distinct bands for NKBOR insertion. However, we do not know the origin of the second band.

The *fimE* gene encodes FimE that along with FimB are the two recombinases, which mediate inversion of the DNA element containing the promoter for *fimA* (Type I fimbriae) [37]. We investigated whether NKBOR insertion into the *fimE* gene altered the level of fimbriae transcription between wild-type and NKBOR *fimE* mutants using qPCR. The level of *fimA* transcript in NKBOR and *DARS1* insertions into *fimE* was increased 7- to 31-fold relative to wild-type (S3A Fig), and remnants of pellicle formation were observed (S3B Fig). This indicates that the fitness advantage linked to loss of *fimE* was due to an increased expression of fimbriae although other yet to be discovered *fimE*-regulated pathways could be involved.

Selection of *DARS1* insertions from a random pool in *DARS1* or *DARS1 DARS2* deficient cells resulted in fewer bands over time on a Southern Blot; i.e., six and five separate band at t = 700, respectively (Fig 2B and 2C). By genome sequencing 71.4% of all *DARS1* insertions in *DARS1* deficient cells mapped to four different insertions in the *fimE* gene (termed *DARS1* Clone *fimE* #1, #2, #3, and #4, Fig 3B, S2 Table). *DARS1* was also inserted in the *ydeS* gene close to *terC* (*DARS1* Clone *ydeS*) and in the intergenic region between *yfbN* and *yfbO* (*DARS1* Clone *yfb*), that of interest has the same gene dosage as the wild-type *DARS1* copy. The functions of putative proteins encoded by *ydeS*, *yfbN* and *yfbO* are not known. Also, *DARS1* in *DARS1* deficient cells was found twice in *yghG* (termed *DARS1* Clone *yghG* #1 and #2) and in *adeQ* (termed *DARS1* Clone *adeQ*). In enterotoxigenic *E*. *coli* YghG is an outer membrane lipoprotein that is required for the correct localization of the GspD secretin in the outer membrane [38], while AdeQ is a high-affinity adenine transporter in *E*. *coli* K-12 [39]. These results indicate that the gene dosage of *DARS1* does not specify its location; i.e., no unique location of *DARS1* was selected for.

Transposon insertions sites for *DARS1* into *DARS1 DARS2* deficient cells were resolved using easy gene walking only. We isolated 20 single clones and mapped their transposon insertion sites. Here, two different insertions were mapped to the *fimE* gene (termed *DARS1* Clone *fimE* #5 and #6, Fig 3C, S3 Table). *DARS1* was also inserted in the intergenic region between *gspA* and *gspC* (*DARS1* Clone *gsp*) and in the intergenic region between *tomB* and *acrB* (*DARS1* Clone *tomB*). *tomB* is in a toxin-antitoxin operon with *hha*, where expression of TomB diminish the toxicity of Hha expression [40]; while the *gspCDEFGHIJKLMO* (*gspC-O*) and *gspAB* operons encode homologs of type II secretion machinery involved in extrusion of folded proteins [41].

Selection of *DARS2* insertions from a random pool in *DARS2* deficient cells resulted in the selection of only one band at t = 700 (Fig 2B), indicating a single optimal chromosomal position for *DARS2*. This correlates well the data above; i.e. movement of *DARS2* had a larger effect on the control of the cell cycle than movement of *DARS1*. Genome sequencing mapped 97.8% of the *DARS2* insertions to two locations approximately 650 bp from the wild-type *DARS2* position, and separated by only 8 bp (S4 Table; Fig 3D). One insertion was located in the intergenic region between the *ptsP* and *rppH* gene (*DARS2* Clone *IR*). This clone was already present at high frequency after 500 generations of growth (S4 Table). The second *DARS2* proximal insertion was located inside the *rppH* gene (*DARS2* Clone *rppH*), which encodes for RppH an RNA pyrophosphohydrolase that initiates mRNA decay [42]. The remaining 2.2% of the mapped inserts were found in the intergenic region between *nanS* and *nanM* (termed *DARS2* Clone *nan*). *nanS* and *nanM* are transcribed in an operon with *nanC*, which supports the efficient use of α-N-acetylneuraminate as the sole source of carbon [43]. The Southern Blot for *DARS2* into *ΔDARS2* showed an abrupt changed between 400 and 500 generations of direct completion (Fig 2B), therefore both time points were resolved by full genome sequencing (S4 Table). Several interesting *DARS2* insertions were found in t = 400, which were not present when the experiment was terminated after 700 generations. One *DARS2* insertion was found in chromosomal position 2.316.202 bp (S4 Table), which is close to the tested *DARS2* mirror position on opposite replication arm (*yafJ*; Table 1). This suggest that these and other may have a fitness advantage over the majority of the initial 70.000 insertions, but that they were overall less fit than the insertions immediately flanking *DARS2* strongly suggesting that the wild-type position is optimal for *DARS2* function.

Two pairs of insertion sites for *DARS2* into Δ*DARS1* Δ*DARS2* were resolved after 700 generations of direct competition. One pair included closely spaced insertions in the *lgoR* gene (*DARS2* Clone *lgoR*) and in the intergenic region between *lgoR* and *lgorT* (*DARS2* Clone IR *lgor*) and the other were two insertions in the gene *ttdR* (*DARS2* Clone *ttdR* #1 and #2) (Fig 3E). *lgoR* is a predicted transcriptional regulator that is essential for growth on L-galactonate as the sole carbon source [44], while *ttdR* is an LysR-type transcriptional regulator for L-tartrate fermentation that is induced under anaerobic growth [45]. The two *DARS2* insertions, *lgoR* and *ttdR*, have almost the same distance to *oriC* albeit located on each replication arm (S5 Table). Thus, indicating that the optimal position of *DARS2* in Δ*DARS1* Δ*DARS2* cells could be linked to gene dosage. The abrupt change between t = 300 and t = 400 for *DARS2* into Δ*DARS1* Δ*DARS2 (*Fig 2C) was also investigated by full genome sequencing (S5 Table). Of interest we find none of the selected end-point at t = 300, but *DARS2* Clone *ttdR* #1, *DARS2* Clone *lgo*, and *DARS2* Clone *lgoR* was found at t = 400.

### Cell cycle and fitness of selected transposon insertions

Representative transposon insertions identified by easy gene walking were moved into a fresh background by P1 transduction and analyzed by flow cytometry (S6 Table). None of the selected clones had a different doubling time compared to wild-type (+/- 2 minutes). All *DARS1* insertions in Δ*DARS1* cells (*fimE* #1, *fimE* #2, *ydeS*, and *yfb*) restored cell cycle parameters to those of wild-type cells, and all *DARS1* insertions in Δ*DARS1* Δ*DARS2* cells (*fimE* #5, *fimE* #6, *gsp*, and *tomB*) restored the phenotype to that of *DARS2* deficient cells. The *DARS2* insertions next to the deleted *DARS2* locus (*IR* and *rppH*) fully complemented loss of *DARS2*. Furthermore, the *DARS2* insertions selected in Δ*DARS1* Δ*DARS2* cells (*lgoR* and *tddR*) restored the low origin/mass ratio to levels close to a cell deficient in only *DARS1*.

We proceeded to investigate the fitness of representative selected clones. Here the *DARS2* Clone *IR*, which was predominant after 700 generations culture evolution, was tested against wild-type and Δ*DARS2* cells. The wild-type was found to be dominant to Δ*DARS2* (1.5 LOG differences after 80 generations of competition) (S4A Fig) as expected [19]. The wild-type was also slightly dominant over the *DARS2* Clone *IR* (0.7 LOG differences) (S4B Fig) whereas *DARS2* Clone *IR* was slightly dominant to Δ*DARS2* (0.8 LOG differences) (S4C Fig). Thus, although *DARS2* Clone *IR* did not have an identical fitness to the wild-type it was more fit than Δ*DARS2* as expected. The pattern seen for *DARS2* Clone *IR* was also observed for *DARS1* Clone *ydeS* tested against the wild-type and Δ*DARS1*, while *DARS1* Clone *fimE* #3 was dominant to both the wild-type and Δ*DARS1*; probably due to the fitness advantage of mutating *fimE* in the used competition experimental setup.

### The activity of *DARS1*, *DARS2*, and *datA* is affected by transcription

**Fig 4:**
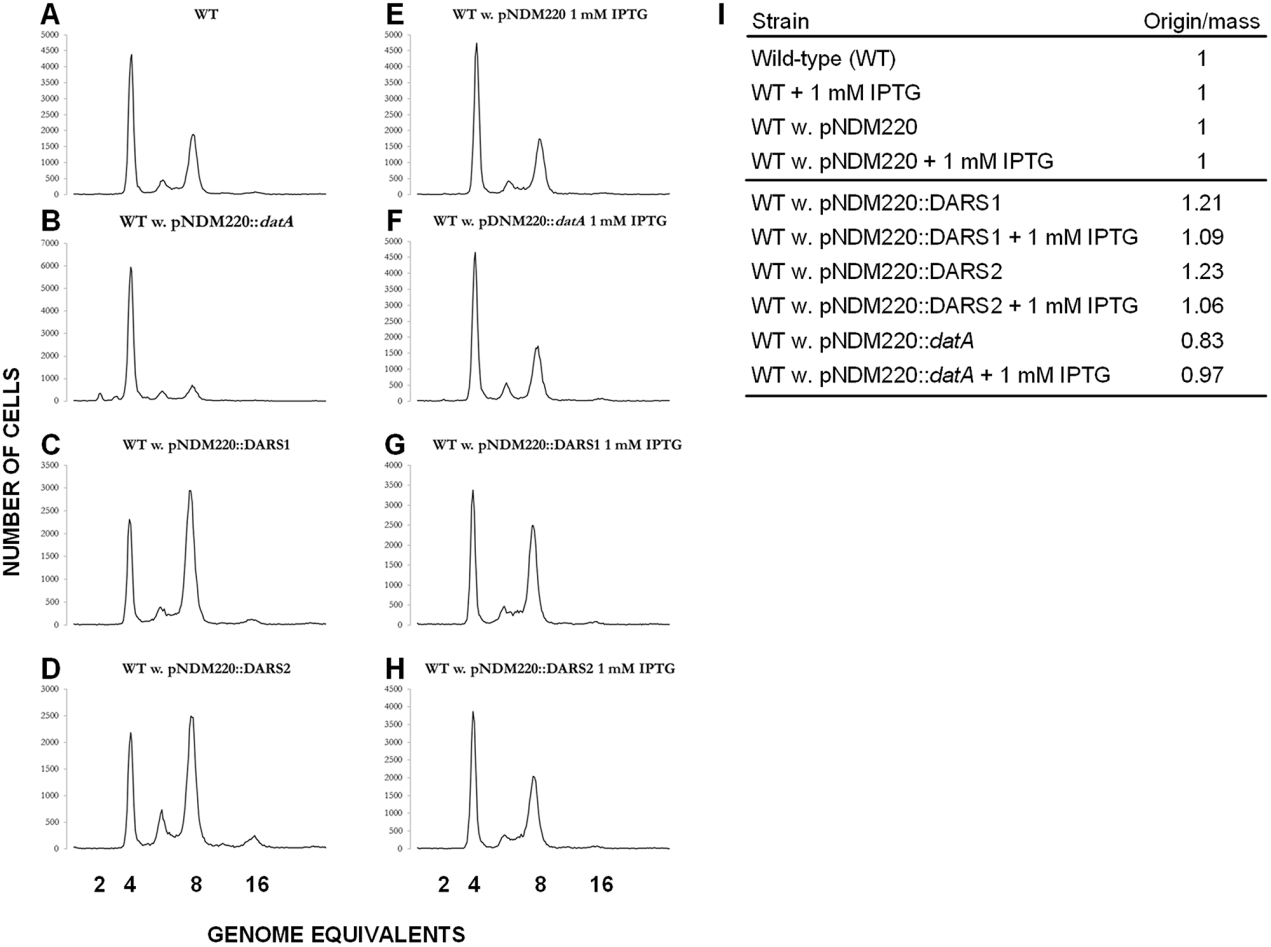
Effect of transcription through *datA*, *DARS1* and *DARS2*. Cells were grown in LB at 32°C with or without 1 mM IPTG. Exponentially growing cells were treated with rifampicin and cephalexin prior to flow cytometric analysis. (A) Wild-type (WT); (B) WT w. pNDM220::*datA*; (C) WT w. pNDM220::*DARS1*; (D) WT w. pNDM220::*DARS2*; (E) WT w. pNDM220 + 1 mM IPTG; (F) WT w. pNDM220::*datA* + 1 mM IPTG; (G) WT w. pNDM220::*DARS1* + 1 mM IPTG; (H) WT w. pNDM220::*DARS2* + 1 mM IPTG. Details are given in the table on the right (I), wherein numbers are normalized to 1 for wild-type.

Gene expression is known to be affected by local chromosomal context [23]. The effect of transcription on activity of cis-acting regions such as *datA*, *DARS1* and *DARS2* is not known. The three regions were cloned into the R1 based plasmid pNDM220 [46], downstream on the IPTG inducible pA1/O4/O3 promoter. The three resultant plasmids were transformed into wild-type cells and their effect on the initiation of replication, in the presence or absence of IPTG, assessed by flow cytometry (Fig 4). The vector plasmid pNDM220 did not alter synchrony or origin concentration irrespective of IPTG addition (compare Fig 4A and 4E). In the absence of IPTG, plasmid pNDM220::*datA* reduced the cellular origin content and concentration without affecting synchrony (compare Fig 4A, 4B and 4I). Plasmid-carried *DARS1* and *DARS2* increased the cellular origin content and concentration (compare Fig 4A and 4C (for *DARS1*), Fig 4D (for *DARS2*), Fig 4I) as previously observed [16]. Only the presence of pNDM220::*DARS2* resulted in asynchronous initiations (Fig 4D).

Strong transcription through either *datA*, *DARS1* or *DARS2*, by addition of IPTG, restored both origin concentration and synchrony to wild-type level (Fig 4F, 4G and 4H). Therefore, transcription through *datA*, *DARS1*, and *DARS2* is detrimental to their function and further enforce that the activity of either region could be affected by local chromosomal context.

## Discussion

We have previously found a conserved distance from *oriC* to the non-coding regions *DARS1*, *DARS2* and *datA* in *E*. *coli* [19]. Site-directed translocation of *DARS1*, *DARS2*, and *datA* showed perturbed regulation of initiation when the regions were relocated away from their wild-type position; highlighting the importance of their natural position.

### Position of *datA*

**Fig 5:**
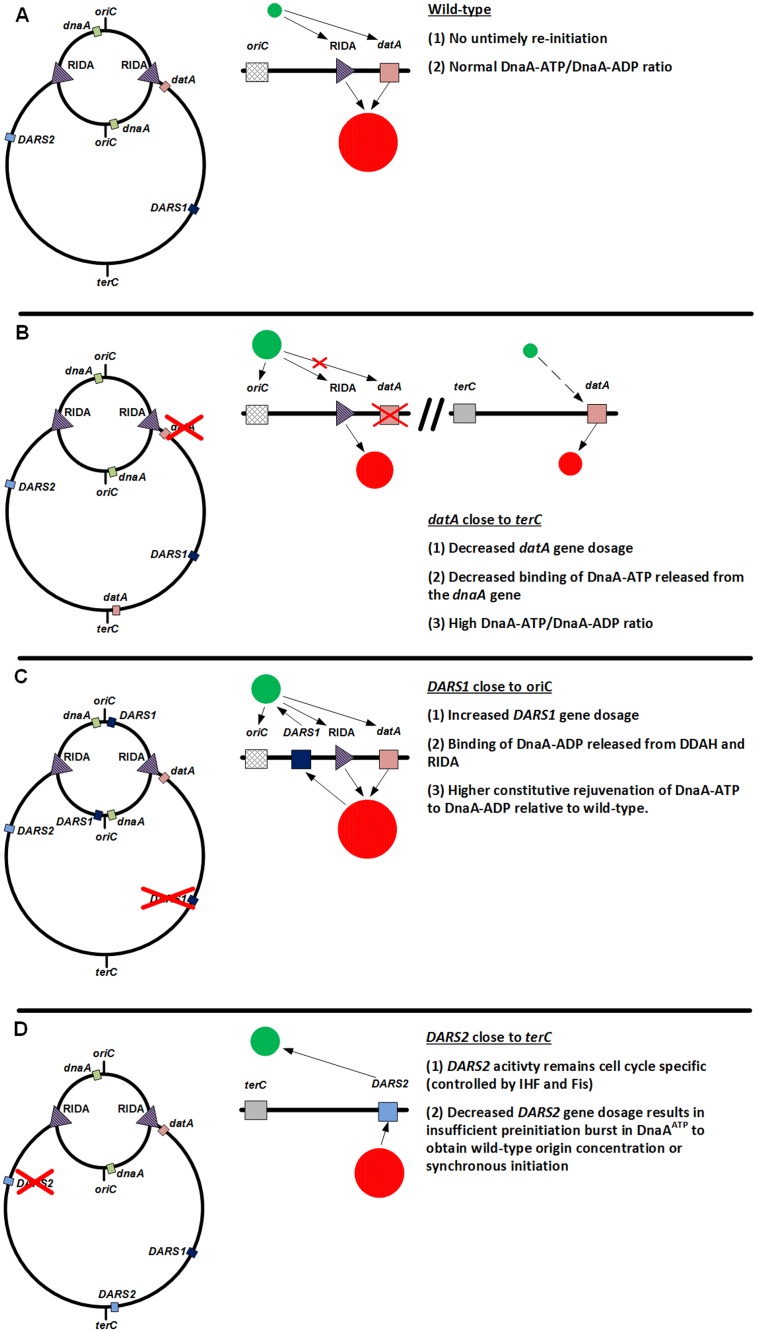
A schematic model of effect of *DARS1* and *datA* relocation. (A) Wild-type; *oriC* and the *dnaA* gene are sequestered by the SeqA/Dam system following initiation of replication ([68]; not on figure). The sequestering period allows RIDA and the early duplication of *datA* to decrease the DnaA^ATP^/DnaA^ADP^ ratio to a level, which counters unwanted re-initiation. (B) Relocation of *datA* close to *terC*; only RIDA and the single copy of *datA* next to *terC* acts to lower the DnaA^ATP^/DnaA^ADP^ ratio during the sequestering period, resulting in higher concentration of DnaA^ATP^ compared to the wild-type. In addition, *datA* is speculated to have increased interaction with DnaA^ATP^ released from the *dnaA* gene due to the close proximity of the two [15]. Therefore the relocation of *datA* to *terC* would lead to decreased inactivation of DnaA^ATP^, a higher DnaA^ATP^/DnaA^ADP^ ratio compared to the wild-type and earlier re-initiation. (C) Relocation of *DARS1* close to *oriC*; RIDA and *datA* acts to lower the DnaA^ATP^/DnaA^ADP^ ratio similar to the wild-type. However, the early duplication of *DARS1* results in an earlier rejuvenation of DnaA^ATP^ from DnaA^ADP^. Thus, with *DARS1* close to *oriC*, the DnaA^ATP^level increases faster han for wild-type cells, which leads to the following initiation at a reduced mass. (D) Relocation of *DARS2* close to *terC*; *DARS2* is cell cycle regulated; i.e. needs to be bound by IHF to be active [17]. IHF will only bind and activate *DARS2* just prior to initiation [17]. Thus, moving *DARS2* close to *oriC* will not alter the origin concentration because IHF availability does not change; i.e. no early rejuvenation activity. However, moving *DARS2* close to *terC* lowers its gene dosage, which may be insufficient to generate the preinitiation burst in DnaA^ATP^ necessary for initiation at all cellular origins; i.e. synchrony. *oriC*, *terC*, *DARS1*, *DARS2*, *datA*, the *dnaA* gene, and RIDA are indicated in the figure. Red circle = DnaA^ADP^, green circle = DnaA^ATP^. The size of the filled green or red circles is proportional to the cellular amounts of DnaA^ATP^ and DnaA^ADP^, respectively.

There are at least two explanations for the over-initiation resulting from *datA* relocation to *terC*; i.e., gene dosage and local access to DnaA^ATP^ (compare Fig 5A to 5B). The chromosomal *datA* position is close to *oriC* and this was proposed to enhance interaction with DnaA^ATP^ released from *oriC* at initiation, resulting in a high DDAH activity [15]. Thus, when *datA* is relocated to *terC*, both gene dosage and interaction with DnaA^ATP^ is diminished resulting in an increased DnaA^ATP^/DnaA^ADP^ ratio and increased origin concentration (Fig 5B). The Gram-positive bacteria *Bacillus subtilis* also contains DnaA box clusters (DBC) that analogue to *datA* can repress untimely initiation [47]. The DBC in *B*. *subtilis* is however, unlike *datA*, only shown to titrate DnaA^ATP^ [47]. Of interest, the genomic position of the DBC is important for regulation of initiation; i.e. relocation from close to *oriC* (wild-type position) to *terC* reduces its function [47], as shown here and previously [28] for *datA*. In addition, we find that the diminished *datA* function in *terC* can be complemented to wild-type by a second *datA* copy in the terminus, suggesting that gene dosage is an important parameter for an optimal *datA* function.

### The optimal positions of *DARS1* and *DARS2*

Directed translocation of *DARS1* indicated little preference for location, and only insertions very close to *oriC* resulted in an increased origin concentration, possibly due to a gene dosage effect or increased interaction with *oriC* associated DnaA (Fig 5C). In agreement with this, culture evolution revealed no optimal chromosomal position for the *DARS1* region, although it´s presence is important [19]. The strong selection for loss of *fimE* in otherwise wild-type cells, suggest that the *DARS1* insertions into *fimE* does not indicate the optimal position of *DARS1* but results from a fitness advantage of disrupting *fimE*. The level of *fimA* transcription was increased in *fimE* mutants relative to the wild-type. As *fimA* encode the Type I major fimbrial subunit, *fimE* mutants are likely to carry an elevated number of fimbria. *fimE* mutant cells formed a pellicle at the top of the culture tubes suggesting, that increased fimbriation facilitates migration towards more aerobic conditions, which in turn could lead to a small growth advantage relative to wild-type cells. The selected *DARS1* insertions into *ydeS*, *yghG*, *adeQ* or *yfb* in *DARS1* deficient cells or in *tomB* or *gsp* in *DARS1 DARS2* deficient cells does not immediately suggest why these locations should provide a fitness advantage, and it may well be that these would be outcompeted by *fimE* insertions had competition proceeded longer. As the majority of the insertions are distant from *oriC*, it may be a fitness disadvantage to have *DARS1* next to the origin.

Directed translocation of *DARS2* indicated that many positions (CAG18499, 18496, 18488, 12135, 18493, and *yafJ*) restored origin concentration and synchrony to wild-type levels. Yet, only two *DARS2* insertions were selected in Δ*DARS2* cells after 700 generations of growth; 97.8% were located next to the wild-type *DARS2* position, while 2.2% were located in the intergenic region between *nanS* and *nanM*. At t = 400, an insertion was found on the opposite replication arm with an almost identical distance to *oriC* as the wild-type *DARS2* position, but this insertion was not recovered after 700 generations. Thus, gene dosage cannot be the single determinant for optimal position. This is corroborated by the inability of a low-copy *DARS2* plasmid to complement *DARS2* deficiency on the chromosome. The present data therefore implies that *DARS2* and the immediate surroundings at its wild-type position specify the optimal genomic position. Even though we only investigate the effects of *DARS2* translocation on the regulation of initiation of replication, we cannot exclude that the optimal *DARS2* position, either directly or indirectly, contributes to fine tune other DnaA functions in the cell.

A cell with two functional *DARS2* regions only resembled wild-type cells when the additional *DARS2* region was located close to *oriC* (high gene dosage) or directly exchanged the wild-type *DARS1* region. This suggests that the local genomic context at *DARS1* also provide an ideal setting also for *DARS2* activity. Interestingly, RNA sequencing reveals very low transcriptional activity at the genomic *DARS1* and *DARS2* locations [48], and we observed that both *DARS1 and DARS2* lost activity by transcription though the regions (Fig 4). Therefore both gene dosage and local transcriptional activity contribute to *DARS* activity. Recent reports also highlight the importance of a conserved genomic position for the function of key regulatory genes [49–51]; i.e. the local genomic environment is important for function. Thus, the importance of the local genomic environment may very well contribute to the selected positions of *DARS1* and *DARS2*.

### *DARS1* and *DARS2* play different roles for replication control

The activity of *DARS1* is neither cell cycle nor growth phase regulated; i.e. always active [16]. Thus, *de novo* synthesis of DnaA will along with the constitutive DnaA^ADP^ rejuvenation at *DARS1* ensure a steady DnaA^ATP^ increase throughout the cell cycle of wild-type cells. Therefore increased *DARS1* gene dosages lead to an increased origin concentration (Fig 5C).

Rejuvenation at *DARS2* is dependent on the binding of both Fis and IHF [17]. Therefore the activity at *DARS2* is growth phase regulated by Fis, i.e. is only active during exponential growth [17], and cell cycle regulated by IHF [17]. Maximal IHF binding to activate *DARS2*, immediately precedes initiation [17]. Initiation of all cellular origins in synchrony was explained by a mechanism where DnaA^ATP^ released from the first origin initiated will trigger initiations on fully methylated not yet initiated origins, by a cascade-like mechanism [52]. However, new initiations will inevitably lead to more DNA loaded β-clamps that are instrumental in RIDA [12] which is therefore expected to accelerate. We propose that timely duplication and activation of *DARS2* raises the DnaA^ATP^/DnaA^ADP^ ratio to a sufficient high level at the onset of initiation, to ensure that all origins present in the cell are initiated in virtual synchrony even though RIDA is increased during the initiation period (for review see [53]). When *DARS2* is located near *terC* the gene dosage may be too low to result in a sufficient pre-initiation burst in DnaA^ATP^ to ensure synchronous initiation at all cellular origins despite of its activity remaining cell cycle regulated (Fig 5D). This explains why a cell with two *DARS2* loci will initiate in synchrony but fail to obtain a wild-type like origin/mass, while a cell with two *DARS1* loci will be asynchronous (Table 2). Thus, *DARS1* is primarily responsible for coupling replication initiation to cell mass increase, whereas *DARS2* is primarily important for maintaining synchronous initiation of all origins contained within a single cell.

*DARS1* and *DARS2*-like sequences (including conserved IHF- and Fis binding sites) with genomic positions similar to *E*. *coli* have been identified in *E*. *coli*-like species [16, 17]. This suggests not only a common mechanism to regulate initiation between species, but also that the genomic position of the regulatory regions are important for correct function in the related species. The variable size of the *E*. *coli* genome, between 4.6 to 5.7 mega base pairs (Mb), indicates that horizontal gene transfer and genome reductions frequently takes place [54]. Thus, its puzzling how new DNA is distributed along the genome to preserve the observed chromosomal symmetry in *E*. *coli* [19], which as shown here at least for *DARS2*, is important for correct fine tuning of initiation and fitness.

## Materials and Methods

### Growth conditions

Cells were grown in Luria–Bertani Broth (LB) medium or AB minimal medium supplemented with 0.2% glucose, 10 μg/ml thiamine, and 0.5% casamino acids. Cells were cultured at 37°C. When necessary, antibiotics were added to the following concentrations: kanamycin, 50 μg/ml; chloramphenicol, 20 μg/ml; ampicillin, 150 μg/ml; tetracycline, 10 μg/ml.

### Bacterial strains

All strains are found in Table 3.

**Table 3:** Bacterial strains.

Strain	Genotype	Abbreviated form	Reference/Source
BW25113	*lacI*^*q*^ *rrnB*_*T14*_ Δl*acZW*_*J16*_ *hsdR514* Δ*araBAD*_*AH33*_ Δ*rhaBAD*_*LD78*_		[55]
MG1655	F^-^λ^-^*rph-1*		[66]
ALO4292	MG1655 str^R^^a^	Wild-type	[19]
ALO4254	Δ*DARS2*::*cat*^a^		[19]
ALO4310	Δ*DARS2*::*cat* str^R^^a^	Δ*DARS2*	[19]
ALO4312	Δ*DARS2* str^R^^a^		[19]
ALO4313	Δ*DARS1*::*cat* str^R^^a^		[19]
ALO4314	Δ*DARS1* str^R^^a^	Δ*DARS1*	This work
ALO4331	Δ*datA*::*kan* str^R^^a^	Δ*datA*	[19]
CAG12135	MG1655 *recD*1901::Tn*10*^a^		[26]
CAG12151	MG1655 *zdh*-925::Tn*10*^a^		[26]
CAG12173	MG1655 *cys*C95::Tn*10*^a^		[26]
CAG18488	MG1655 *zjd*-2231::Tn*10*^a^		[26]
CAG18493	MG1655 *zbi*-29::Tn*10*^a^		[26]
CAG18496	MG1655 *fadAB*101::Tn*10*^a^		[26]
CAG18499	MG1655 *zid*-501::Tn*10*^a^		[26]
ALO4412	Δ*DARS1* CAG18499::*DARS1*::*cat* tetR strR^a^		This work
ALO4413	Δ*DARS1* CAG18499::*DARS2*::*cat* tetR strR^a^		This work
ALO4414	Δ*DARS2* CAG18499::*DARS1*::*cat* tetR strR^a^		This work
ALO4415	Δ*DARS2* CAG18499::*DARS2*::*cat* tetR strR^a^		This work
ALO4475	Δ*datA* CAG18499::*datA*::*cat* tetR strR^a^		This work
ALO4440	Δ*DARS1* CAG18496::*DARS1*::*cat* tetR strR^a^		This work
ALO4441	Δ*DARS1* CAG18496::*DARS2*::*cat* tetR strR^a^		This work
ALO4442	Δ*DARS2* CAG18496::*DARS1*::*cat* tetR strR^a^		This work
ALO4443	Δ*DARS2* CAG18496::*DARS2*::*cat* tetR strR^a^		This work
ALO4476	Δ*datA* CAG18496::*datA*::*cat* tetR strR^a^		This work
ALO4378	Δ*DARS1* CAG18488::*DARS1*::*cat* tetR strR^a^		This work
ALO4379	Δ*DARS1* CAG18488::*DARS2*::*cat* tetR strR^a^		This work
ALO4380	Δ*DARS2* CAG18488::*DARS1*::*cat* tetR strR^a^		This work
ALO4381	Δ*DARS2* CAG18488::*DARS2*::*cat* tetR strR^a^		This work
ALO4477	Δ*datA* CAG18488::*datA*::*cat* tetR strR^a^		This work
ALO4585	Δ*DARS1* CAG12135::*DARS1*::*cat* tetR strR^a^		This work
ALO4586	Δ*DARS1* CAG12135::*DARS2*::*cat* tetR strR^a^		This work
ALO4463	Δ*DARS2* CAG12135::*DARS1*::*cat* tetR strR^a^		This work
ALO4464	Δ*DARS2* CAG12135::*DARS2*::*cat* tetR strR^a^		This work
ALO4419	Δ*DARS1* CAG12173::*DARS1*::*cat* tetR strR^a^		This work
ALO4420	Δ*DARS1* CAG12173::*DARS2*::*cat* tetR strR^a^		This work
ALO4421	Δ*DARS2* CAG12173::*DARS1*::*cat* tetR strR^a^		This work
ALO4478	Δ*datA* CAG12173::*datA*::*cat* tetR strR^a^		This work
ALO4431	Δ*DARS1* CAG18493::*DARS1*::*cat* tetR strR^a^		This work
ALO4432	Δ*DARS1* CAG18493::*DARS2*::*cat* tetR strR^a^		This work
ALO4433	Δ*DARS2* CAG18493::*DARS1*::*cat* tetR strR^a^		This work
ALO4434	Δ*DARS2* CAG18493::*DARS2*::*cat* tetR strR^a^		This work
ALO4479	Δ*datA* CAG18493::*datA*::*cat* tetR strR^a^		This work
ALO4390	Δ*DARS1* CAG12151::*DARS1*::*cat* tetR strR^a^		This work
ALO4608	Δ*DARS1* CAG12151::*DARS2*::*cat* tetR strR^a^		This work
ALO4391	Δ*DARS2* CAG12151::*DARS1*::*cat* tetR strR^a^		This work
ALO4392	Δ*DARS2* CAG12151::*DARS2*::*cat* tetR strR^a^		This work
ALO4480	Δ*datA* CAG12151::*datA*::*cat* tetR strR^a^		This work
ALO4328	Δ*DARS1* CAG18461::*DARS1*::*cat* tetR strR^a^		This work
ALO4329	Δ*DARS1* CAG18461::*DARS2*::*cat* tetR strR^a^		This work
ALO4591	Δ*DARS2* CAG18461::*DARS1*::*cat* tetR strR^a^		This work
ALO4330	Δ*DARS2* CAG18461::*DARS2*::*cat* tetR strR^a^		This work
ALO4481	Δ*datA* CAG18461::*datA*::*cat* tetR strR^a^		This work
ALO5041	Δ*datA* CAG12151::*datA* CAG18461::*datA*::*cat* tetR strR^a^		This work
ALO5042	Δ*DARS2 yafJ*::*DARS2*::*cat* strR^a^		This work
ALO5043	Δ*DARS1*::*DARS2*::*cat* strR^a^		This work
ALO4255	MG1655 *tonA*		This work
ALO4256	Δ*DARS1*::*cat tonA*^a^		This work
ALO4257	Δ*DARS2*::*cat tonA*^a^		This work
ALO4259	Δ*DARS1*::*cat* Δ*DARS2 tonA*^a^		This work

^a^ Genotype otherwise as *E*. *coli* MG1655

### Plasmids

#### Plasmids for movement of *DARS1*, *DARS2*, and *datA*

The chromosomal *DARS1*, *DARS2*, and *datA* locus were PCR amplified from MG1655 using primers *DARS1*_pKD3_FW and *DARS1*_pKD3_RV, *DARS2*_pKD3_FW and *DARS2*_pKD3_RV, and *datA*_pKD3_FW and *datA*_pKD3_RV (S7 Table), respectively. The PCR products were cut with *Not*I and *Pvu*I and ligated with *Not*I- and *Pvu*I-cut pKD3 [55], resulting in pKD3::*DARS1* (pJFM5), pKD3::*DARS2* (pJFM6), and pKD3::*datA* (pJFM9). Each plasmid was confirmed by sequencing.

#### *DARS2* on low-copy number plasmid

The chromosomal *DARS2* region was PCR amplified from MG1655 using primers *DARS2*_pALO277_FW and *DARS2*_pALO277_RV (S7 Table). The resultant PCR products was cut with *Sal*I and *Bst*BI and ligated with *Sal*I- and *Bst*BI cut pALO277 [30], resulting in pALO277::*DARS2* (pJFM7). The plasmid was confirmed by sequencing.

#### Plasmids for transposon-mediated random insertion of *DARS1* and *DARS2*

The chromosomal *DARS1* and *DARS2* loci were PCR amplified from MG1655 using primers *DARS1*_pNKBOR_FW and *DARS1*_pNKBOR_RV and *DARS2*_pNKBOR_FW and *DARS2*1_pNKBOR_RV (S7 Table), respectively. The resultant PCR products were cut with *Bam*HI and *Pst*I and ligated with *Bam*HI- and *Pst*I cut pNKBOR [35]. Each PCR product was inserted between the two IS*10* IR elements, resulting in NKBOR::*DARS1* (pJFM3) and NKBOR::*DARS2* (pJFM1). Each plasmid was confirmed by sequencing.

#### Plasmids for investigating the effect of transcription through *DARS1*, *DARS2*, and *datA*

The chromosomal DARS1, DARS2, and *datA* locus was PCR amplified from MG1655 using primers DARS1_pNDM220_FW and DARS1_pNDM220_RV, DARS2_pNDM220_FW and DARS2_pNDM220_RV, and *datA*_pNDM220_FW and *datA*_pNDM220_RV (S7 Table), respectively. The PCR products were cut with *Bam*HI and *Eco*RI and ligated with *Bam*HI- and *Eco*RI-cut pNDM220 [46], resulting in pNDM220::DARS1 (pJFM10), pNDM220::DARS2 (pJFM11), and pNDM220::*datA* (pJFM12). Each plasmid was confirmed by sequencing.

### Relocation of *datA*, *DARS1*, and *DARS2*

A set of strains containing the transposon Tn*10* (encoding tetracycline resistance) at known positions on the chromosome were described previously [27] and generously provided by Dr. Martin G. Marinus (Fig 1A). None of the Tn*10* insertions from the Singer library altered origin concentration (origins/mass), synchrony of initiation of replication, or doubling time of otherwise wild-type cells.

The Tn*10* insertions CAG18499, CAG18496, CAG18488, CAG12173, CAG1315, CAG18493, CAG12151, and CAG18461 were individually moved into BW25113 [55], MG1655 str^R^ (ALO4292), MG1655 str^R^ Δ*DARS1* (ALO4314), MG1655 str^R^ Δ*DARS2* (ALO4312), and MG1655 str^R^ Δ*datA*::*kan* (ALO4331)[19] by P1 transduction using established procedures [56] and by selection for tetracycline resistance.

In the individual Tn*10* constructed strains the *tetC* gene on the Tn*10* was replaced by either the *datA*, *DARS1*, or *DARS2* region linked to the *cat* gene by the lambda red procedure [55]. Briefly, *datA* was PCR amplified from pJFM9, *DARS1* from pJFM5, and *DARS2* from pJFM6 using primers *tetC*_FW and *tetC*_RV (S7 Table). *tetC*_FW and *tetC_*RV will PCR amplify both the cloned locus into pKD3 (see above) along with the *cat* cassette including the two FRT-sties.

CAG18499::*datA*::*cat*, CAG18496::*datA*::*cat*, CAG18488::*datA*::*cat*, CAG12173::*datA*::*cat*, CAG18493::*datA*::*cat*, CAG18461::*datA*::*cat*, and CAG12151::*datA*::*cat* were individually moved from BW25113 into MG1655 strR Δ*datA*::*kan* by P1 transduction and by selection for *cat* and *kan* resistance. Hence, derivatives of the wild-type strain devoid of *datA* at the original locus and instead carrying Tn*10*::*datA* at indicated chromosomal loci were created. In addition, a strain with two Tn*10*::*datA* loci in the terminus region were created by removing the *cat* gene from CAG18461::*datA*::*cat*, by pCP20, according to a method described previously [57]. Thereafter the CAG12151::*datA*::*cat* were moved from BW25113 into MG1655 strR Δ*datA*::*kan* CAG18461::*datA* by P1 transduction, selecting for *cat* resistance, resulting in a strain with *datA* inserted in both CAG18461 and CAG12151 (ALO5041).

CAG18499::*DARS1*::*cat*, CAG18496::*DARS1*::*cat*, CAG18488::*DARS1*::*cat*, CAG12135::*DARS1*::*cat*, CAG12173::*DARS1*::*cat*, CAG18493::*DARS1*::*cat*, and CAG18461::*DARS1*::*cat*, CAG12151::*DARS1*::*cat* were individually moved from BW25113 into MG1655 str^R^ Δ*DARS1* and MG1655 str^R^ Δ*DARS2* by P1 transduction and by selection for *cat* resistance. Hence, derivatives of the wild-type strain devoid of *DARS1* at the original locus and instead carrying Tn*10*::*DARS1* at indicated chromosomal loci were created (Table 2). Similarly, derivatives of the wild-type strain devoid of *DARS2* at the original locus and instead carrying Tn*10*::*DARS1* at indicated chromosomal loci were created.

CAG18499::*DARS2*::*cat*, CAG18496::*DARS2*::*cat*, CAG18488::*DARS2*::*cat*, CAG12135::*DARS2*::*cat*, CAG18493::*DARS2*::*cat*, CAG18461::*DARS2*::*cat*, and CAG12151::*DARS2*::*cat* were individually moved from BW25113 into MG1655 str^R^ Δ*DARS1* and MG1655 str^R^ Δ*DARS2* by P1 transduction and by selection for *cat* resistance. Hence, derivatives of the wild-type strain devoid of *DARS2* at the original locus and instead carrying Tn*10*::*DARS2* at indicated chromosomal loci were created (Table 2). Also derivatives of the wild-type strain devoid of *DARS1* at the original locus and instead carrying Tn*10*::*DARS2* at indicated chromosomal loci were created. Furthermore, *DARS2* were relocated to *yafJ*. *yafJ* was replaced with *DARS2* amplified from pJFM6 using primers *yafJ*_FW and *yafJ*_RV (S7 Table) in MG1655 StrR *ΔDARS2* harboring pKD46, resulting in the MG1655 StrR *ΔDARS2 yafJ*::*DARS2*::*cat* mutant (ALO5042). *DARS2* was also relocated to *DARS1* in MG1655 StrR. *DARS1* was replaced with *DARS2* amplified from pJFM6 using primers *D2toD1*_FW and *D2toD1*_RV (S7 Table) in MG1655 StrR harboring pKD46, resulting in the MG1655 StrR *ΔDARS1*::*DARS2*::*cat* mutant (ALO5043).

### Transposon-mediated random insertion of *DARS1* and *DARS2*

A spontaneous *tonA* mutant of *E*. *coli* MG1655 was isolated by Dr. Stanley Brown, resulting in *E*. *coli* MG1655 *tonA* (ALO4255).

The *DARS1* region was replaced with the *cat* gene in MG1655 harboring pKD46, resulting in the Δ*DARS1*::*cat* mutant (ALO4075). Briefly, the *cat* gene was PCR amplified using primers *DARS1*_KO_FW and *DARS1*_KO_RV from pKD3. The resultant DNA fragments were introduced into ALO1825 bearing pKD46. Each deletion was verified by PCR. The *DARS1* deletion was moved from ALO4075 to ALO4255 by P1 transduction, selecting for chloramphenicol resistance, resulting in MG1655 *tonA* Δ*DARS1*::*cat* (ALO4256). The *DARS2* deletion was moved from ALO4254 into ALO4255 by P1 transduction, selecting for chloramphenicol resistance, resulting in MG1655 *tonA* Δ*DARS2*::*cat* (ALO4257). The *cam* cassette was removed from ALO4257 and the *DARS1* deletion was moved from ALO4075 into the chloramphenicol sensitive MG1655 *tonA* Δ*DARS2* by P1 transduction, selecting for chloramphenicol resistance, resulting in MG1655 *tonA* Δ*DARS1*::*cat* Δ*DARS2* (ALO4259).

### Replication-associated gene dosage

The replication-associated gene dosage was estimated for every position for growth in ABTG + CAA and LB. Eq (1) was used to calculate the replication-associated gene dosage Here the average no. of copies per chromosome of a gene with position x on the chromosome (x = 0 at the origin and x = 1 at the terminus), C is the replication period in minutes, D is the time following termination of replication until cell division, and τ is the doubling time [58].

$$X=\;2^{\left(\frac{\left[C\;\times \;\left(1-x\right)\;+D\right]}{\tau }\right)}\;$$ (1)

$$G=\;\frac{\tau }{C\;\times \text{ln}\left(2\right)}\left[2^{\frac{C+D}{\tau }}-2^{D/\tau }\right]$$ (2)

At generation times below 60 min the C- and D period has been shown to be constant in *E*. *coli* K-12 strains; i.e. 42 minutes and 33 minutes, respectively [59]. Generation time for the wild-type grown in ABTG + CAA was shown to be 40 minutes. By Eq (1) the gene dosage of *DARS2* is calculated to be 1.66 copies/cell at the given growth rate. Hence, by Eq (2), the cellular DNA content (G) was found to be 1.82 genome equivalents per cell. As the copy number of plasmid F is 1–2 per genome equivalent [31] it follows that the copy number per cell of the F plasmid pALO277 and derivatives is 1.82–3.64.

### In culture competition experiment for transposon-mediated random insertion of *DARS1* and *DARS2*

pNKBOR is a R6K-based suicide vector that permits the random insertion of a mini-transposon (NKBOR) into a π protein deficient *E*. *coli* chromosome [35]. Here we transformed pJFM1 (NKBOR::*DARS2*) into Δ*DARS2* and Δ*DARS1* Δ*DARS2*, pJFM3 (NKBOR::*DARS2*) into Δ*DARS1* and Δ*DARS1* Δ*DARS2*, and pNKBOR (NKBOR) into wild-type selecting for *kan* resistance. Approximately 70,000 random insertions were obtained for each transformation. The 70,000 strains were pooled and inoculated in the same tube. They were grown in LB aerated by continuous shaking at 37°C. The populations were propagated by continuously transfers after estimated 10 generations. Samples for genomic DNA from each population were taken at 100-generation intervals, until direct competition for estimated 700-generations (used for southern blot; Fig 2). After 700-generations of direct competition 10 single clones was isolated from NKBOR::*DARS2* into Δ*DARS2*, 20 single clones were isolated from NKBOR::*DARS1* into Δ*DARS1*, from NKBOR::*DARS1* into Δ*DARS1* Δ*DARS2*, and from NKBOR::*DARS2* into Δ*DARS1* Δ*DARS2*, and 15 single clones were isolated from NKBOR into wild-type.

### Southern blot analysis

Total cellular DNA was prepared according to Løbner-Olesen and von Freiesleben [60]. DNA was digested with *Pvu*I, and fragments were separated on a 0.7% agarose gel, transferred by capillary transfer to a Hybond-N^+^ membrane (Amersham Pharmacia Biotech), and probed with an approx. 1 kb NKBOR fragment, which hybridize to NKBOR. The probe was prepared by PCR amplification using primers NKBOR_Probe_FW and NKBOR_Probe_RV (S7 Table) using pNKBOR as template and labeled with [α-^32^P]dATP (Amersham Pharmacia) using the Random Primer system (DECAprime II DNA Labeling Kit; Life Technologies).

### Easy gene walking

The chromosomal position of the NKBOR, NKBOR::*DARS1*, and NKBOR::*DARS2* insertion was found as described by Harrison *et al*. [36], using nested primers specific to NKBOR (NKBOR_N3, NKBOR_N2, and NKBOR_1) and random primers Random_*Hind*III and Random_*Sau*3AI (S7 Table).

### Whole-genome sequencing

Whole-genome sequencing was performed at the University of Copenhagen on an Illumina MiSeq benchtop sequencer. A total of 6 million paired-end reads were generated, with read length of 35 to 300 nucleotides. Reads were aligned to 150 N’s contiguous to NKBOR, AF310136.1 1,904…2,204 using Bowtie2 [61] with interval between seed substrings = S,1,1.15 and maximum number of ambiguous characters = L,0,0.9. Aligned reads were then aligned to MG1655 ref|NC_000913.3 and NKBOR gb|AF310136 using blastN [62] and sorted for contiguous alignment MG1655-NKBOR. Note that the coverage in the present deep sequencing was insufficient for a complete mapping of insertion sites. The data presented in S1–S5 Tables therefore contains a representative subset of the total number of insertions.

### RNA isolation and cDNA synthesis

Total RNA from bacterial samples was extracted using the GeneJET RNA Purification Kit (Thermo Fisher Scientific) according to the manual. Following treatment of RNA with TURBO DNase (Ambion), cDNA was synthesized using the RevertAid First Strand cDNA Synthesis Kit for reverse transcriptase PCR according to the manufacturer’s protocols (Thermo Fisher Scientific). In parallel, RNA samples were subjected to agarose gel electrophoresis and NanoDrop (Thermo Fisher Scientific) to verify quality and yield. qPCR with primers specific to the *rpoA* gene (the α subunit of the RNA polymerase core enzyme) (S7 Table) [63] was performed on cDNA samples prepared with and without reverse transcriptase to confirm no genomic DNA contamination of the RNA preparations following DNase treatment.

### Quantitative polymerase chain reaction (qPCR)

#### fimA

Primers designed to amplify *fimA* (S7 Table) were targeted to regions of unique sequence within the gene. The qPCR was performed using Takara SYBR Premix Ex Taq II (RR820A) in a BioRAD CFX96 (95°C 30 s, 39×(95°C 5 s + 60°C 30 s), 95°C 15 s, 60°C 60 s). All data were normalized to the endogenous reference gene *rpoA* [63] with primers taken from the same article. These data were transformed to log2 to obtain a change difference (n-fold) between strains.

#### F plasmid to *ter*

F plasmid copy number quantified relative to *ter* was performed as previously described [64].

### Relative distance

The relative distance between *oriC* and transposon insertions sites were calculated as described previously [19].

### Flow cytometry

Flow cytometry was performed as described previously [65] using an Apogee A10 instrument. For each sample, a minimum of 30.000 cells were analyzed. Numbers of origins per cell and relative cell mass were determined as described previously [65].

### Asynchrony index

Asynchrony was calculated as described by Løbner-Olesen *et al*. [65]. Initiations were considered asynchronous when A>0.1.

### Competition experiment in LB

The fitness of *DARS2* Clone *rrpH*, *DARS1* Clone *fimE* #3, and *DARS1* Clone *ydeS* were compared to the wild-type and either *ΔDARS1* or *ΔDARS2* (indicated in the text) during direct competition in LB medium. The competing strains were inoculated pairwise at an approximate concentration of (10^7^ CFU/mL) each. The populations were propagated by continuously transfers in LB medium. Samples from each population were taken at 10-generation intervals. Each sample was diluted in 0.9% NaCl and plated on LB plates with appropriate antibiotics. All plates were incubated for 18–24h at 37°C prior to counting.

## Supporting Information

S1 Fig Effect of *DARS2* complementation on a low-copy number plasmid. (A) The *ter*/F ratio was determined by qPCR from wild-type cell with either pALO277 or pALO277::*DARS2* (as indicated). B-G: Flow cytometric analysis of wild-type and *DARS2* deficient cells with or without the plasmids. Cells were grown in AB minimal medium supplemented with 0.2% glucose, 10 μg/ml thiamine, and 0.5% casamino acids at 37°C. Wild-type (MG1655) or Δ*DARS2* carried no plasmid, pALO277, or pALO277:*DARS2* as indicated in individual panels. Details are given in the table on the right (H), wherein numbers are normalized to 1 for wild-type.
(TIF)

S2 Fig Representative flow cytometry histograms of *DARS* complementation. Representative flow cytometry histograms of *DARS1*/*DARS2* insertions close to *oriC* (CAG18499), a Tn*10* insertion close to *datA* (CAG18488), and one close to *terC* (CAG12151). Cells were grown in AB minimal medium supplemented with 0.2% glucose, 10 μg/ml thiamine, and 0.5% casamino acids at 37°C. Wild-type, Δ*DARS1*, and Δ*DARS2* are shown in A, B, and F, respectively. Derivatives of the wild-type strain MG1655 devoid of *DARS1* at the original locus and instead carrying an additional copy of *DARS2* close to *oriC*, close to *datA*, or close to *terC* are shown in C, D, and E, respectively. Derivatives of the wild-type strain MG1655 devoid of *DARS2* at the original locus and instead carrying an additional copy of *DARS1* close to *oriC*, close to *datA*, or close to *terC* are shown in G, H, and I, respectively. See Table 2 for details.
(TIF)

S3 Fig Insertions in *fimE* leads to increased fimbriation. (A) Quantification of the *fimA* mRNA level. Quantitative PCR was performed as described in Materials and Methods. Relative *fimA* mRNA levels in strains Δ*DARS1*, Δ*DARS1* Δ*DARS2*, NKBOR Clone *fimE*, *DARS1* Clone *fimE* #1, *DARS1* Clone *fimE* #2, and *DARS1* Clone *fimE* #3 were determined. In this experiment, the *rpoA* mRNA was used as an internal control. Three biological measurements were performed, and standard deviations are shown. (B) Remnants of pellicle formation. Wild-type (1.) and NKBOR Clone *fimE* (2.) grown for 10 hours in LB at 37C. Framed is the presumed remnant from the pellicle in NKBOR Clone *fimE*. The culture had been shaking (diagonally) in a shaker overnight. Thus when hold diagonally the pellicle remnant would align with the surface of the culture. The picture was however taken with the tubes more or less hold vertical to better show the difference in pellicle formation between fimE^+^ (wild-type) and fimE^-^ (NKBOR Clone *fimE*).
(TIF)

S4 Fig Fitness of selected *DARS2* insertions. Competition experiment in LB medium. A: Wild-type vs. Δ*DARS2*; B: Wild-type vs *DARS2* Clone *rppH*; C: Δ*DARS2* vs *DARS2* Clone *rppH*. Bars represent the standard error of the log10 mean number of CFU per ml. For details see Supporting Information.
(TIF)

S1 Table NKBOR into wild-type. ^a^ Chromosomal position of transposon insertion site on the *E*. *coli* MG1655 genome a t = 0 resolved by full genome sequencing. Percent of aligned reads is given in the brackets. A total of 50 insertions were resolved for this time point. ^b^ Chromosomal position of transposon insertion site on the *E*. *coli* MG1655 genome a t = 700 resolved by full genome sequencing. Percent of aligned reads is given in the brackets. A total of 228 insertions were resolved for this time point.
(TIF)

S2 Table *DARS1* into Δ*DARS1*. ^a^ Chromosomal position of transposon insertion site on the *E*. *coli* MG1655 genome a t = 0 resolved by full genome sequencing. Percent of aligned reads is given in the brackets. A total of 14 insertions were resolved for this time point. ^b^ Chromosomal position of transposon insertion site on the *E*. *coli* MG1655 genome a t = 700 resolved by full genome sequencing. Percent of aligned reads is given in the brackets. A total of 227 insertions were resolved for this time point.
(TIF)

S3 Table *DARS1* into Δ*DARS1* Δ*DARS2*. ^a^ Chromosomal position of transposon insertion site on the *E*. *coli* MG1655 genome a t = 700 resolved by easy gene walking.
(TIF)

S4 Table *DARS2* into Δ*DARS2*. ^a^ Chromosomal position of transposon insertion site on the *E*. *coli* MG1655 genome a t = 0 resolved by full genome sequencing. Percent of aligned reads is given in the brackets. A total of 39 insertions were resolved for this time point. ^b^ Chromosomal position of transposon insertion site on the *E*. *coli* MG1655 genome a t = 400 resolved by full genome sequencing. Percent of aligned reads is given in the brackets. A total of 54 insertions were resolved for this time point. ^c^ Chromosomal position of transposon insertion site on the *E*. *coli* MG1655 genome a t = 500 resolved by full genome sequencing. Percent of aligned reads is given in the brackets. A total of 13 insertions were resolved for this time point. ^d^ Chromosomal position of transposon insertion site on the *E*. *coli* MG1655 genome a t = 700 resolved by full genome sequencing. Percent of aligned reads is given in the brackets. A total of 278 insertions were resolved for this time point.
(TIF)

S5 Table *DARS2* into Δ*DARS1* Δ*DARS2*. ^a^ Chromosomal position of transposon insertion site on the *E*. *coli* MG1655 genome a t = 0 resolved by full genome sequencing. Percent of aligned reads is given in the brackets. A total of 9 insertions were resolved for this time point. ^b^ Chromosomal position of transposon insertion site on the *E*. *coli* MG1655 genome a t = 300 resolved by full genome sequencing. Percent of aligned reads is given in the brackets. A total of 20 insertions were resolved for this time point. ^c^ Chromosomal position of transposon insertion site on the *E*. *coli* MG1655 genome a t = 400 resolved by full genome sequencing. Percent of aligned reads is given in the brackets. A total of 52 insertions were resolved for this time point. ^d^ Chromosomal position of transposon insertion site on the *E*. *coli* MG1655 genome a t = 700 resolved by full genome sequencing. Percent of aligned reads is given in the brackets. A total of 275 insertions were resolved for this time point.
(TIF)

S6 Table Flow cytometric characterizing of NKBOR, NKBOR::*DARS1*, and NKBOR::*DARS2* insertions. ^a^ Transposon sites found be Easy-Gene Walking at t = 700. ^b^ The transposon insertions selected from the five set-ups were moved into a fresh background by P1 transduction; NKBOR Clone *fimE* into wild-type, *DARS1* Clone *fimE* #1, *fimE* #2, *ydeS*, and *yfb* into *DARS1* deficient cells, *DARS1* Clone *fimE* #5, *fimE* #6, *tomB*, and *gsp* into *DARS1 DARS2* deficient cells, *DARS2* Clone IR and *rppH* into *DARS2* deficient cells, and *DARS2* Clone *tddR* and *lgoR* into *DARS1 DARS2* deficient cells. ^c^ Doubling time in LB grown at 37°C. ^d^ Determined as average light scatter from flow cytometric analysis. Numbers are normalized to 1 for wild-type. ^e^ Asynchrony index; calculated as described in Methods.
(TIF)

S7 Table Primers. (TIF)
