# Supplementary figures and images for: DNA Replication Control Is Linked to Genomic Positioning of Control Regions in Escherichia coli
Source: PLoS Genet. 2016 Sep 2;12(9):e1006286. doi: 10.1371/journal.pgen.1006286 (PMC5010248; doi:10.1371/journal.pgen.1006286)

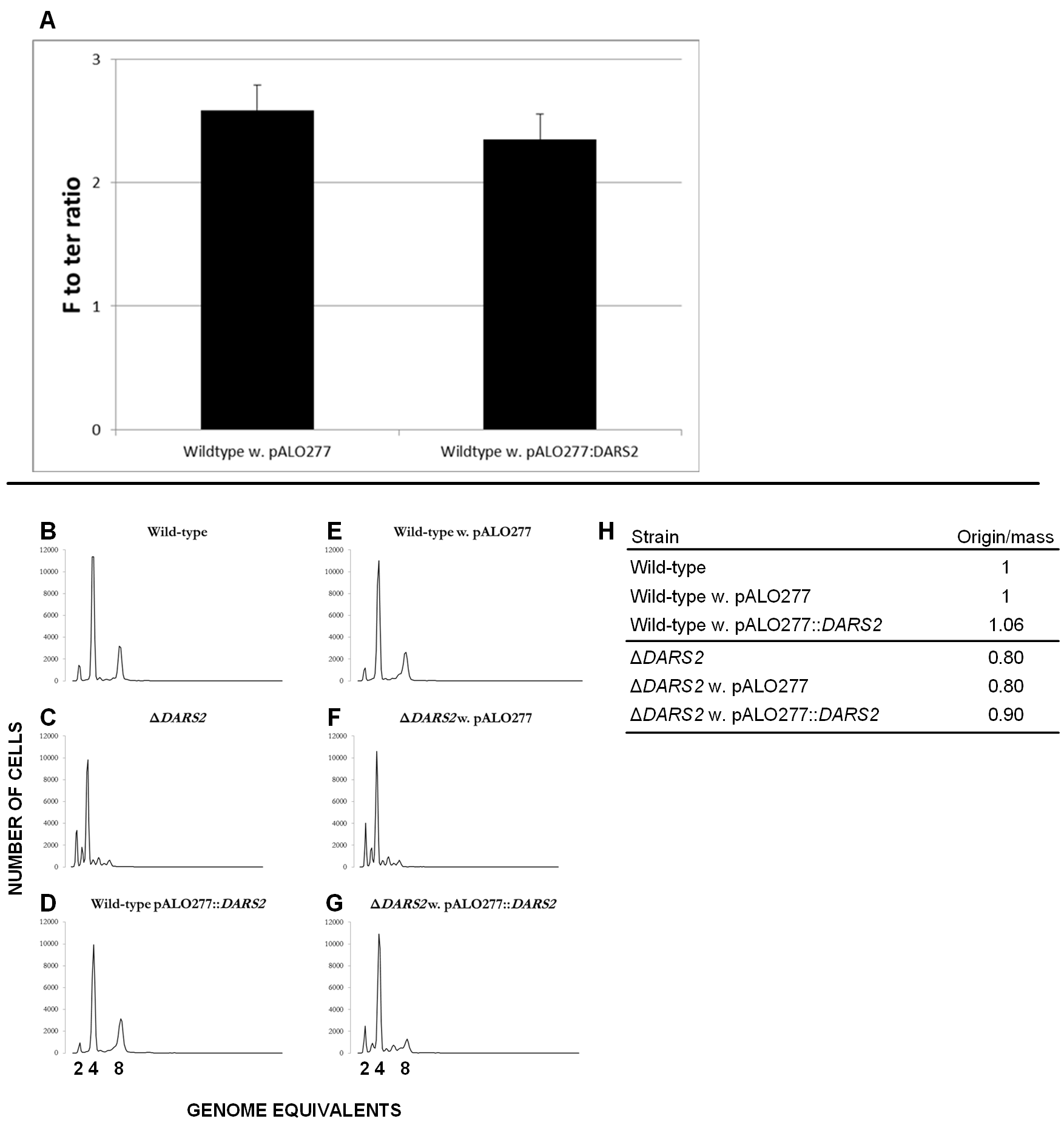

Supplement: S1 Fig — (A) The ter/F ratio was determined by qPCR from wild-type cell with either pALO277 or pALO277::DARS2 (as indicated). B-G: Flow cytometric analysis of wild-type and DARS2 deficient cells with or without the plasmids. Cells were grown in AB minimal medium supplemented with 0.2% glucose, 10 μg/ml thiamine, and 0.5% casamino acids at 37°C. Wild-type (MG1655) or ΔDARS2 carried no plasmid, pALO277, or pALO277:DARS2 as indicated in individual panels. Details are given in the table on the right (H), wherein numbers are normalized to 1 for wild-type. (TIF) [file pgen.1006286.s001.tif]

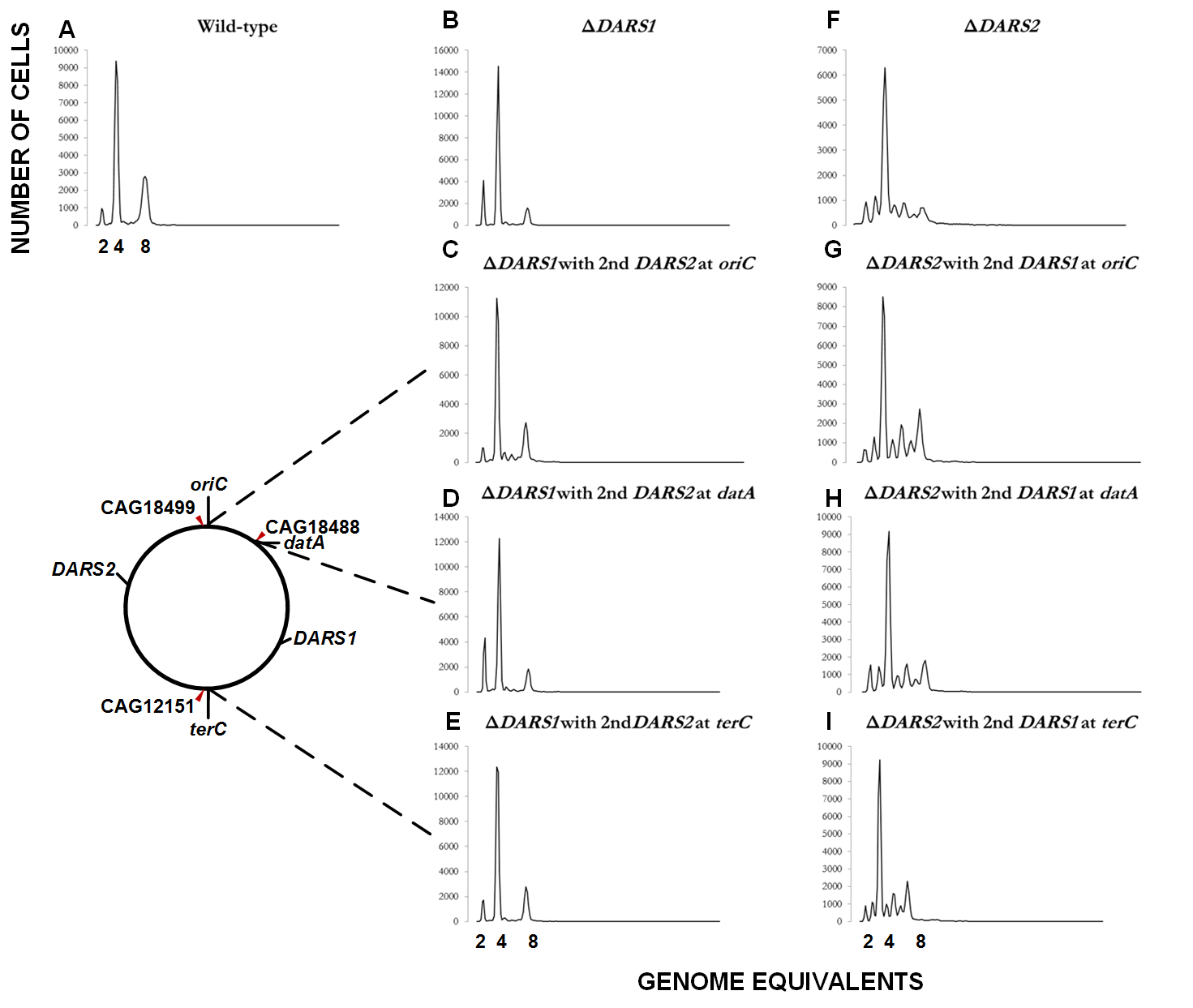

Supplement: S2 Fig — Representative flow cytometry histograms of DARS1/DARS2 insertions close to oriC (CAG18499), a Tn10 insertion close to datA (CAG18488), and one close to terC (CAG12151). Cells were grown in AB minimal medium supplemented with 0.2% glucose, 10 μg/ml thiamine, and 0.5% casamino acids at 37°C. Wild-type, ΔDARS1, and ΔDARS2 are shown in A, B, and F, respectively. Derivatives of the wild-type strain MG1655 devoid of DARS1 at the original locus and instead carrying an additional copy of DARS2 close to oriC, close to datA, or close to terC are shown in C, D, and E, respectively. Derivatives of the wild-type strain MG1655 devoid of DARS2 at the original locus and instead carrying an additional copy of DARS1 close to oriC, close to datA, or close to terC are shown in G, H, and I, respectively. See Table 2 for details. (TIF) [file pgen.1006286.s002.tif]

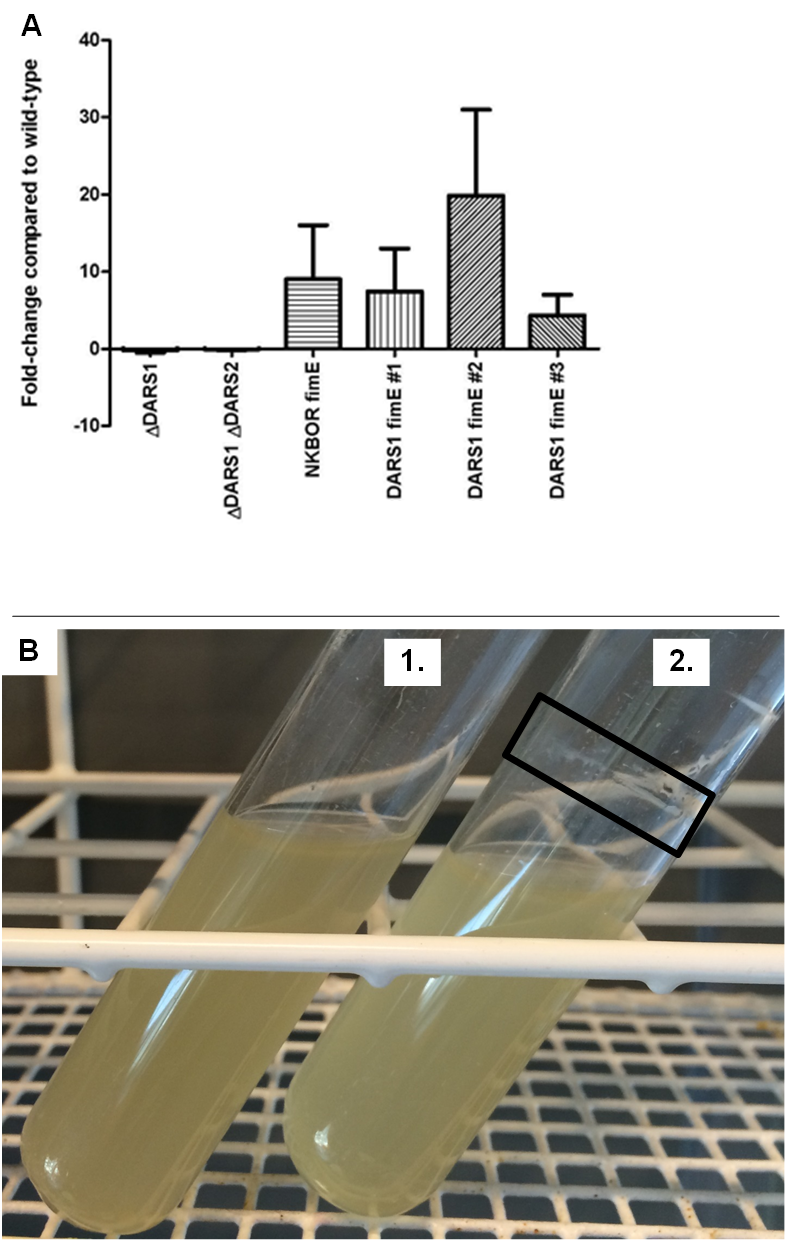

Supplement: S3 Fig — (A) Quantification of the fimA mRNA level. Quantitative PCR was performed as described in Materials and Methods. Relative fimA mRNA levels in strains ΔDARS1, ΔDARS1 ΔDARS2, NKBOR Clone fimE, DARS1 Clone fimE #1, DARS1 Clone fimE #2, and DARS1 Clone fimE #3 were determined. In this experiment, the rpoA mRNA was used as an internal control. Three biological measurements were performed, and standard deviations are shown. (B) Remnants of pellicle formation. Wild-type (1.) and NKBOR Clone fimE (2.) grown for 10 hours in LB at 37C. Framed is the presumed remnant from the pellicle in NKBOR Clone fimE. The culture had been shaking (diagonally) in a shaker overnight. Thus when hold diagonally the pellicle remnant would align with the surface of the culture. The picture was however taken with the tubes more or less hold vertical to better show the difference in pellicle formation between fimE+ (wild-type) and fimE- (NKBOR Clone fimE). (TIF) [file pgen.1006286.s003.tif]

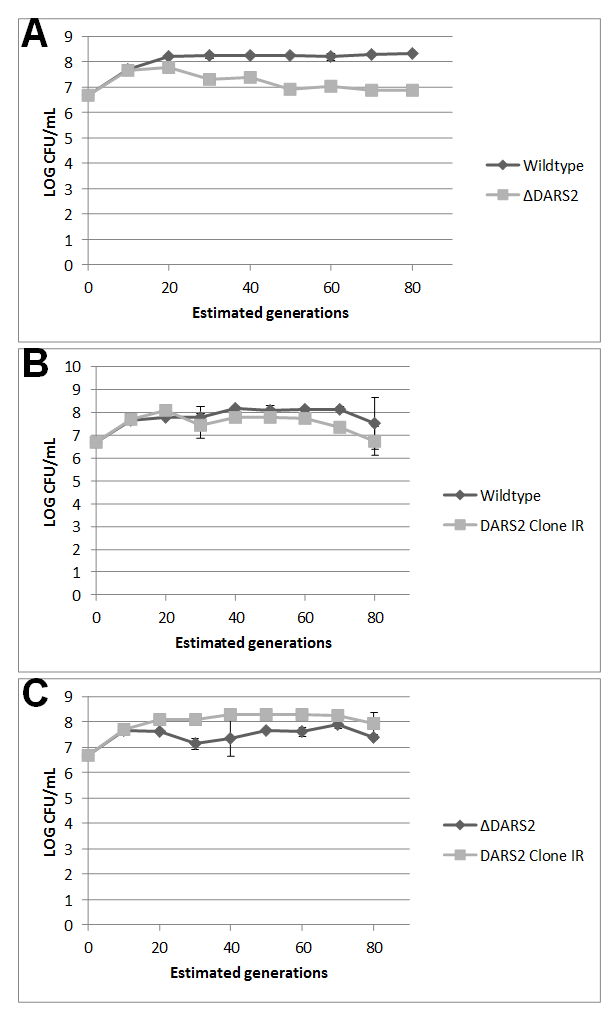

Supplement: S4 Fig — Competition experiment in LB medium. A: Wild-type vs. ΔDARS2; B: Wild-type vs DARS2 Clone rppH; C: ΔDARS2 vs DARS2 Clone rppH. Bars represent the standard error of the log10 mean number of CFU per ml. For details see Supporting Information. (TIF) [file pgen.1006286.s004.tif]

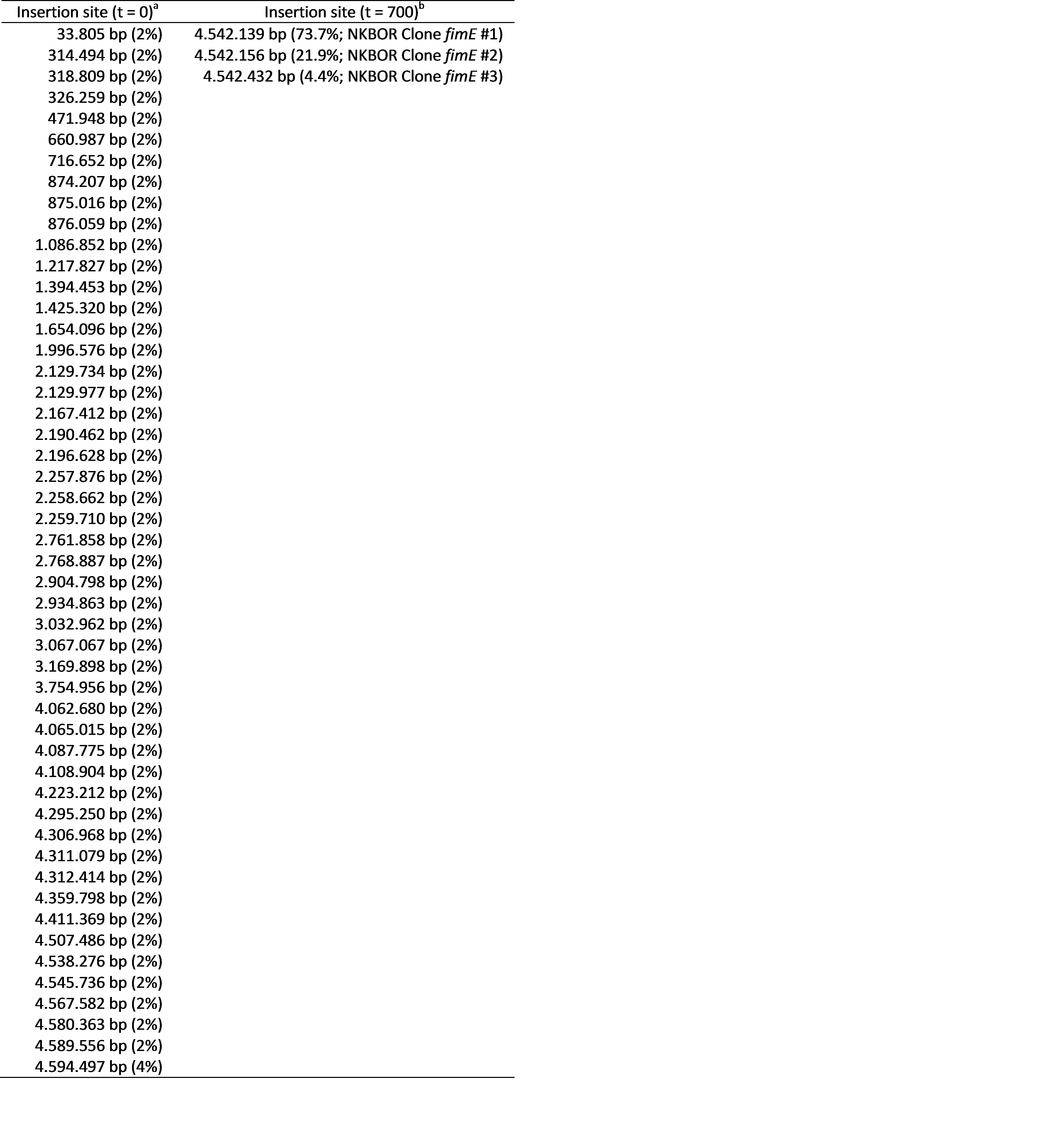

Supplement: S1 Table — a Chromosomal position of transposon insertion site on the E. coli MG1655 genome a t = 0 resolved by full genome sequencing. Percent of aligned reads is given in the brackets. A total of 50 insertions were resolved for this time point. b Chromosomal position of transposon insertion site on the E. coli MG1655 genome a t = 700 resolved by full genome sequencing. Percent of aligned reads is given in the brackets. A total of 228 insertions were resolved for this time point. (TIF) [file pgen.1006286.s005.tif]

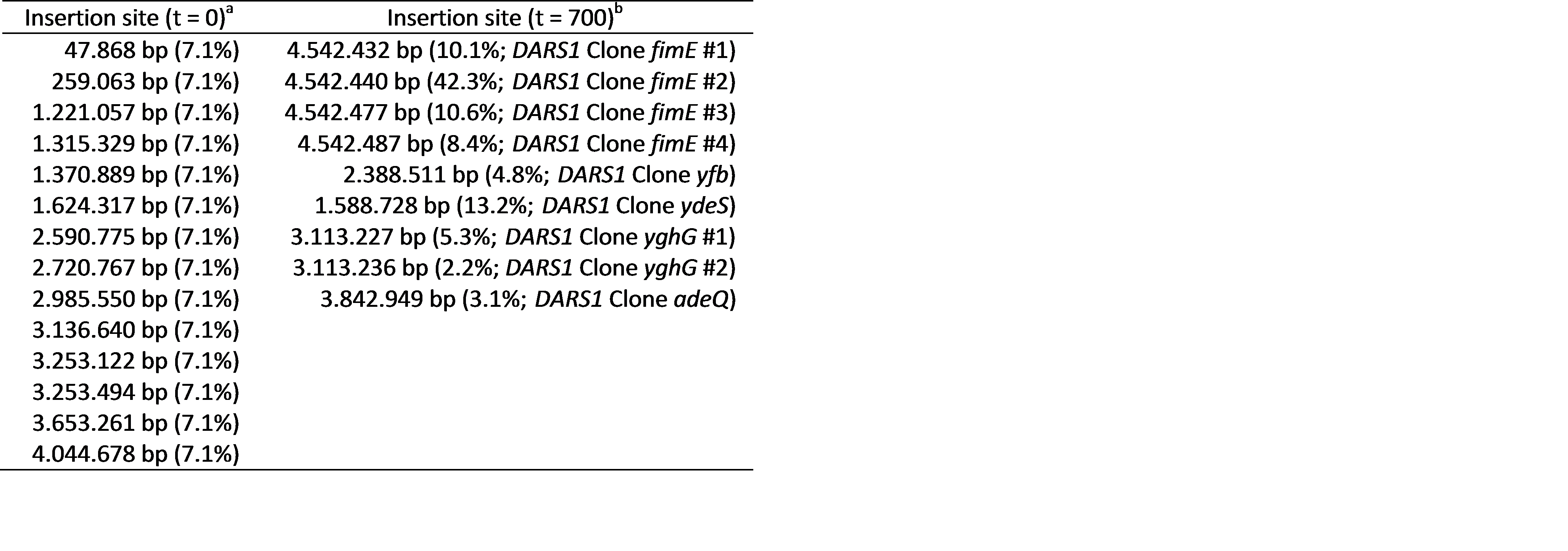

Supplement: S2 Table — a Chromosomal position of transposon insertion site on the E. coli MG1655 genome a t = 0 resolved by full genome sequencing. Percent of aligned reads is given in the brackets. A total of 14 insertions were resolved for this time point. b Chromosomal position of transposon insertion site on the E. coli MG1655 genome a t = 700 resolved by full genome sequencing. Percent of aligned reads is given in the brackets. A total of 227 insertions were resolved for this time point. (TIF) [file pgen.1006286.s006.tif]

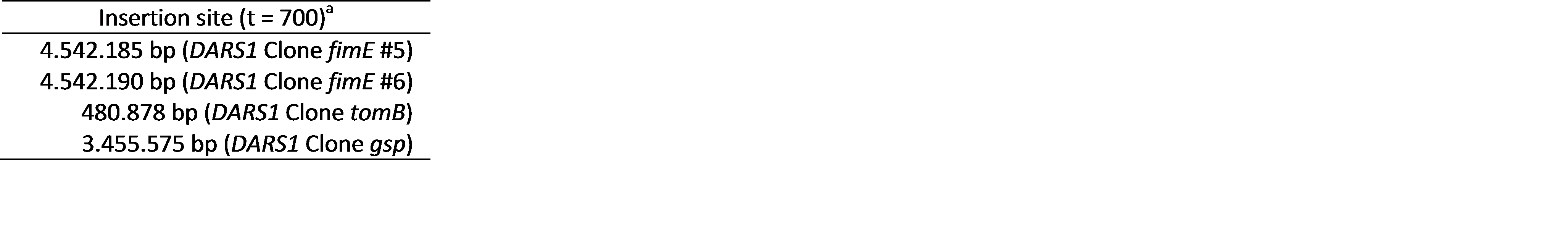

Supplement: S3 Table — a Chromosomal position of transposon insertion site on the E. coli MG1655 genome a t = 700 resolved by easy gene walking. (TIF) [file pgen.1006286.s007.tif]

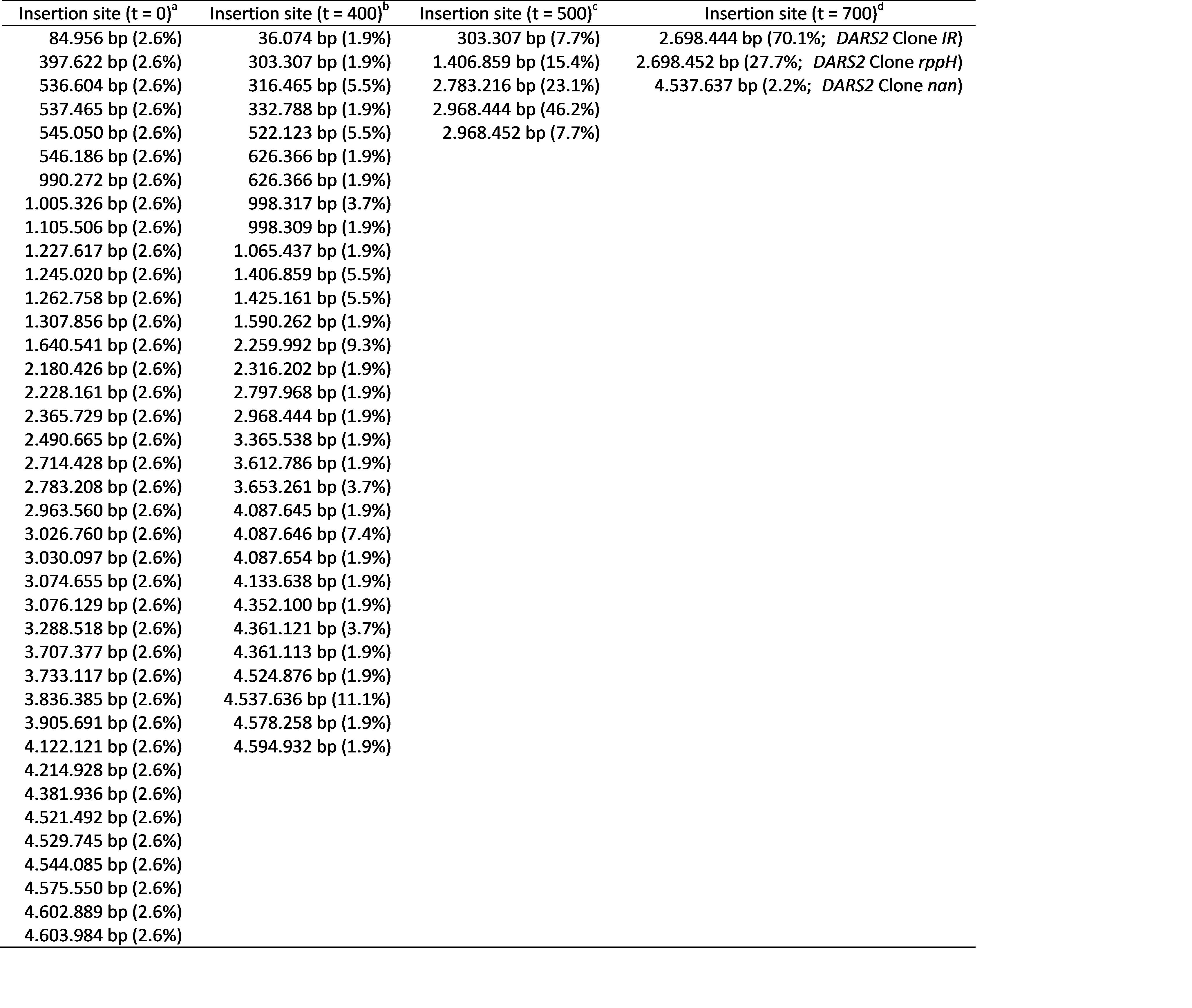

Supplement: S4 Table — a Chromosomal position of transposon insertion site on the E. coli MG1655 genome a t = 0 resolved by full genome sequencing. Percent of aligned reads is given in the brackets. A total of 39 insertions were resolved for this time point. b Chromosomal position of transposon insertion site on the E. coli MG1655 genome a t = 400 resolved by full genome sequencing. Percent of aligned reads is given in the brackets. A total of 54 insertions were resolved for this time point. c Chromosomal position of transposon insertion site on the E. coli MG1655 genome a t = 500 resolved by full genome sequencing. Percent of aligned reads is given in the brackets. A total of 13 insertions were resolved for this time point. d Chromosomal position of transposon insertion site on the E. coli MG1655 genome a t = 700 resolved by full genome sequencing. Percent of aligned reads is given in the brackets. A total of 278 insertions were resolved for this time point. (TIF) [file pgen.1006286.s008.tif]

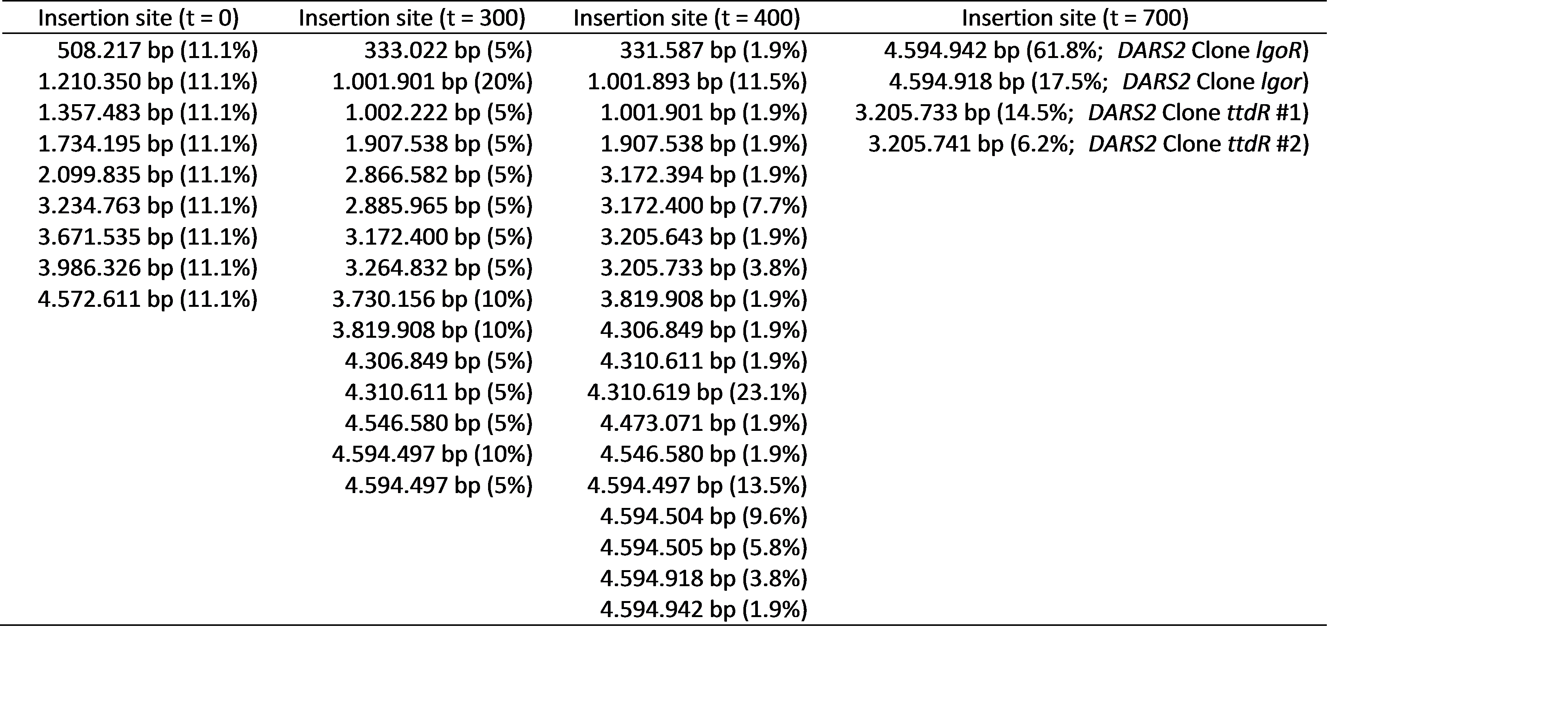

Supplement: S5 Table — a Chromosomal position of transposon insertion site on the E. coli MG1655 genome a t = 0 resolved by full genome sequencing. Percent of aligned reads is given in the brackets. A total of 9 insertions were resolved for this time point. b Chromosomal position of transposon insertion site on the E. coli MG1655 genome a t = 300 resolved by full genome sequencing. Percent of aligned reads is given in the brackets. A total of 20 insertions were resolved for this time point. c Chromosomal position of transposon insertion site on the E. coli MG1655 genome a t = 400 resolved by full genome sequencing. Percent of aligned reads is given in the brackets. A total of 52 insertions were resolved for this time point. d Chromosomal position of transposon insertion site on the E. coli MG1655 genome a t = 700 resolved by full genome sequencing. Percent of aligned reads is given in the brackets. A total of 275 insertions were resolved for this time point. (TIF) [file pgen.1006286.s009.tif]

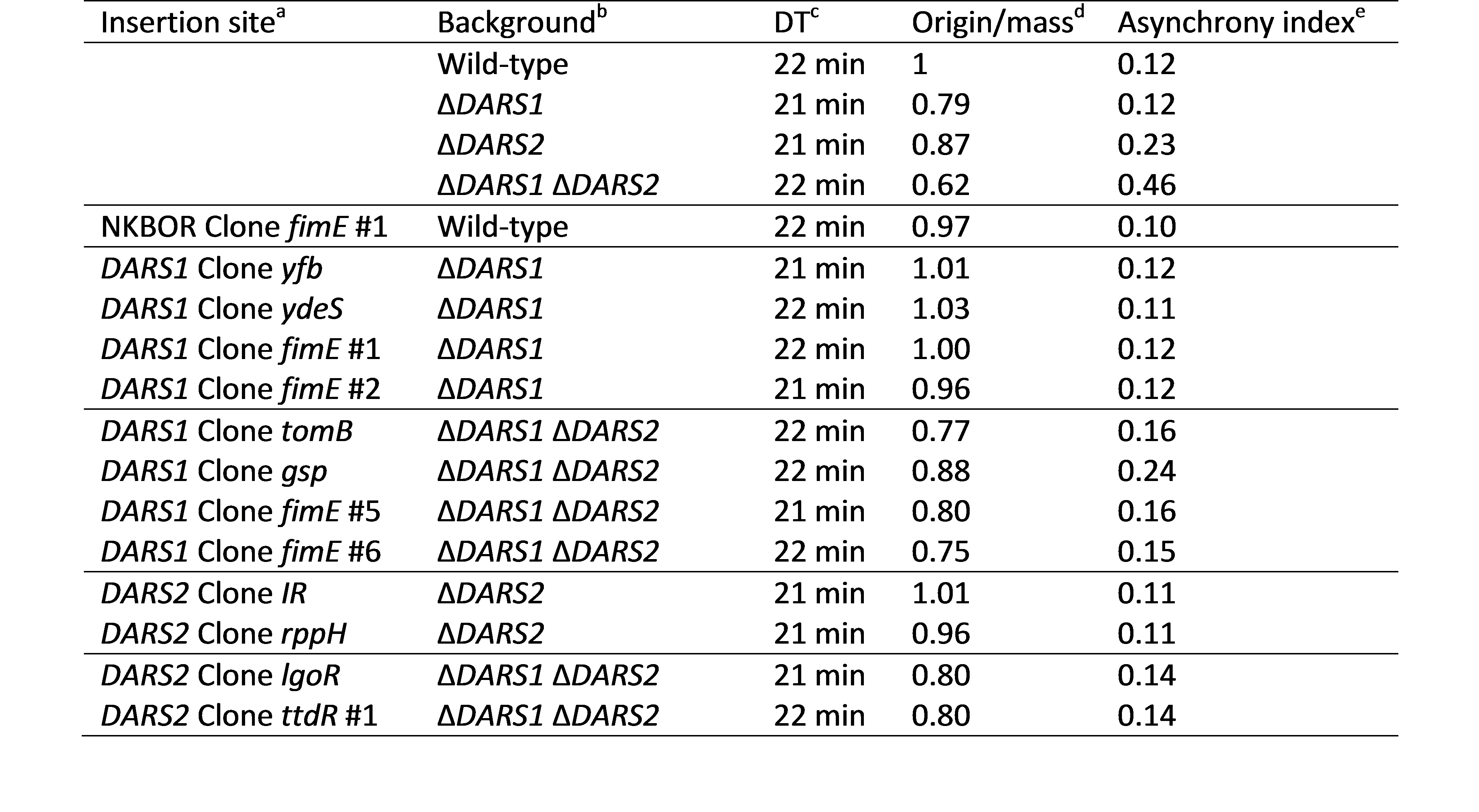

Supplement: S6 Table — a Transposon sites found be Easy-Gene Walking at t = 700. b The transposon insertions selected from the five set-ups were moved into a fresh background by P1 transduction; NKBOR Clone fimE into wild-type, DARS1 Clone fimE #1, fimE #2, ydeS, and yfb into DARS1 deficient cells, DARS1 Clone fimE #5, fimE #6, tomB, and gsp into DARS1 DARS2 deficient cells, DARS2 Clone IR and rppH into DARS2 deficient cells, and DARS2 Clone tddR and lgoR into DARS1 DARS2 deficient cells. c Doubling time in LB grown at 37°C. d Determined as average light scatter from flow cytometric analysis. Numbers are normalized to 1 for wild-type. e Asynchrony index; calculated as described in Methods. (TIF) [file pgen.1006286.s010.tif]

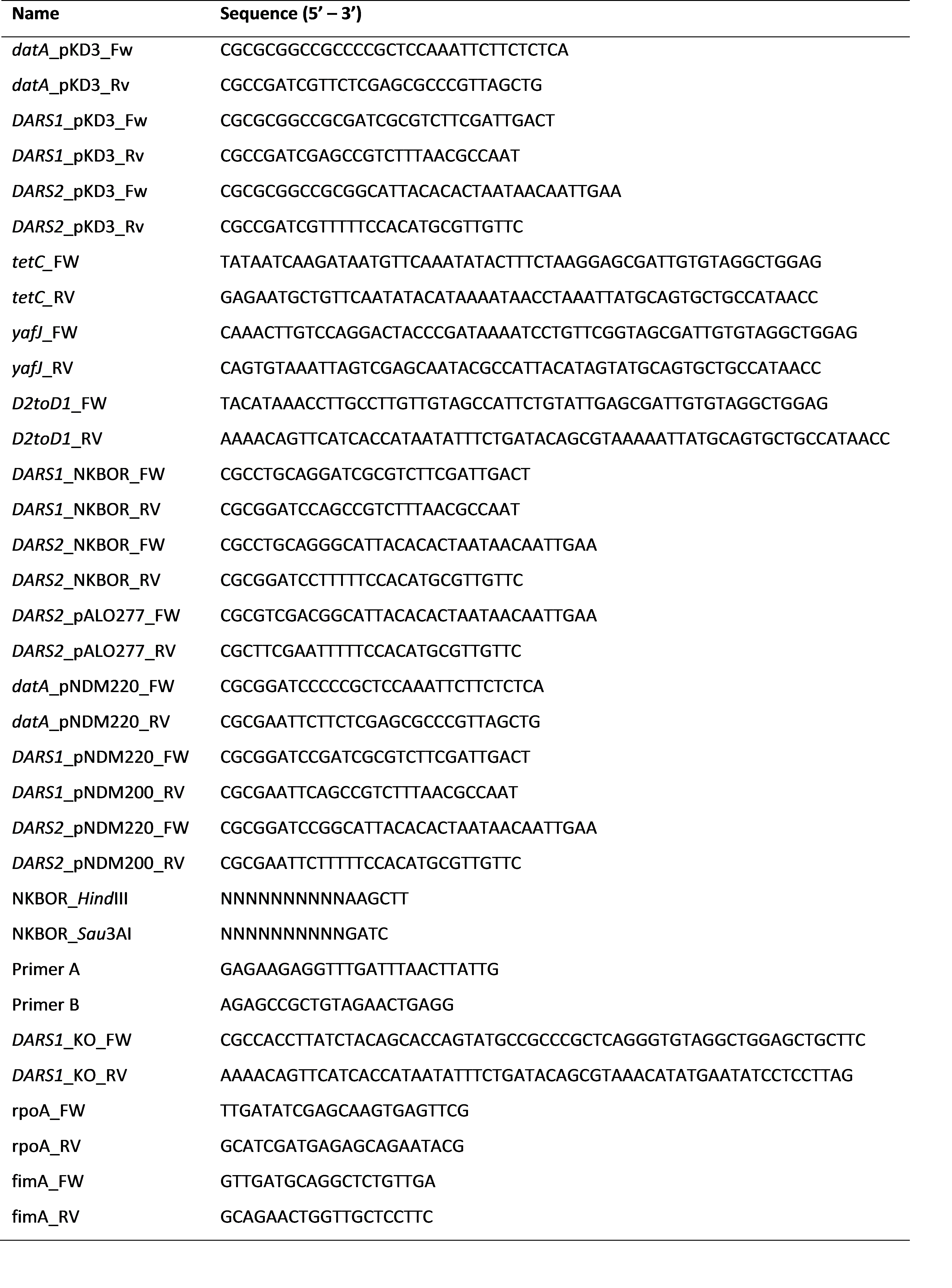

Supplement: S7 Table — (TIF) [file pgen.1006286.s011.tif]
